# Black phosphorus nanosheet-promoted prodrug activation for enhanced cancer therapy

**DOI:** 10.1016/j.mtbio.2026.103244

**Published:** 2026-05-17

**Authors:** Nan Yang, Dabao Zha, Changyu Cao, Ruigang Liu, Hanjun Sun, Xuejiao Song, Yanling Li, Xiaoji Xie, Xiaochen Dong, Yu Cai

**Affiliations:** a Center for Rehabilitation Medicine, Rehabilitation & Sports Medicine Research Institute of Zhejiang Province, Department of Rehabilitation Medicine, Cancer Center, Zhejiang Provincial People's Hospital (Affiliated People's Hospital), Hangzhou Medical College, Hangzhou, 310014, China; b State Key Laboratory of Flexible Electronics (LoFE) & Institute of Advanced Materials (IAM), School of Flexible Electronics (Future Technologies), Nanjing Tech University (NanjingTech), Nanjing, 211816, China; c School of Chemistry & Materials Science, School of Physics and Electronic Engineering, Jiangsu Normal University, Xuzhou, 221116, China

**Keywords:** Black phosphorus, Prodrug, Hypoxia, Tumor, Cancer therapy

## Abstract

Tumor hypoxia-activated prodrugs possess great potential for cancer therapy. However, their therapeutic effects are still limited by several factors, including suboptimal conversion to the active pharmaceutical ingredient in complex tumor environments. Here, we demonstrate a strategy using black phosphorus nanosheets (BPNSs) to boost the activation and therapeutic effects of a tumor hypoxia-activated prodrug, banoxantrone dihydrochloride (AQ4N). BPNSs can facilitate the activation of AQ4N to a toxic anticancer drug through their degradation, which consumes oxygen and acidifies the surrounding environment, and their selective interaction with AQ4N under slightly acidic conditions, which can activate AQ4N. Moreover, the combination of BPNSs and AQ4N can result in synergistic anticancer effects as an additional benefit. Using the features of BPNSs, an enhanced cancer therapy without obvious toxic side effects was achieved using AQ4N in a mouse model.

## Highlights

• BPNSs promote AQ4N activation under acidic and hypoxic tumor conditions.
• BPNS degradation consumes O_2_ and acidifies tumors to boost prodrug efficacy.
• BPNSs plus AQ4N suppress tumors in vivo with minimal systemic toxicity.

## Graphical abstract

**Figure:**
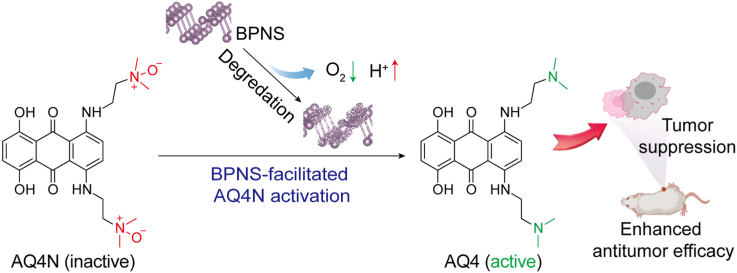

## 1 Introduction

Low oxygen (O_2_) concentration (i.e., the O_2_ partial pressure) in tumors, known as tumor hypoxia, is prevalent in solid tumors and represents a typical characteristic of most advanced solid tumors [1]. Generally, tumor hypoxia results from the disequilibrium between inadequate O_2_ supply and excessive O_2_ consumption caused by the rapid proliferation of cancer cells. In solid tumors, hypoxia can activate transcription factors, particularly hypoxia-inducible factor (HIF), to reprogram various cellular processes, including cell metabolism, cell cycle progression, angiogenesis, and protein biosynthesis, enabling cancer cells to survive and proliferate under hypoxic circumstances [2]. Moreover, hypoxia impairs the efficacy of clinically used anticancer treatments, including chemotherapy, radiotherapy, and photodynamic therapy, resulting in unfavorable tumor prognosis [[3], [4], [5], [6], [7], [8], [9], [10], [11]]. Consequently, targeting and exploiting tumor hypoxia represents a compelling strategy for achieving desired anticancer outcomes. In parallel, tumor microenvironment (TME) modulation has emerged as a promising direction in nanomedicine, with various stimuli-responsive nanoplatforms designed to respond to or remodel TME features such as hypoxia, acidity, and redox imbalance for enhanced therapeutic efficacy [[12], [13], [14], [15]].

Exploiting the hypoxia characteristic of solid tumors, hypoxia-activated prodrugs (HAPs) have been designed and have emerged as selective and effective anticancer agents [[16], [17], [18]]. HAPs typically contain a trigger moiety that undergoes one-electron reduction to form radical anions. These radical anions can undergo further reduction to generate cytotoxic effector species under hypoxic conditions but are neutralized by O_2_ in a futile redox cycle, thereby enabling selective anticancer treatment [19]. To date, HAPs bearing different trigger moieties, including aromatic N-oxides/nitro groups, quinones, and cobalt complexes, have been developed. Among them, tirapazamine and banoxantrone dihydrochloride (AQ4N) are encouraging anticancer agents; however, the heterogeneous and dynamic nature of tumor hypoxia limits their therapeutic efficacy [[20], [21], [22]]. To overcome these challenges, strategies including O_2_ depletion and disruption of O_2_ supply have been developed to exacerbate tumor hypoxia. These strategies typically employ composites of HAPs and auxiliary materials to enhance therapeutic efficacy. Nevertheless, the accumulation of such composites in non-target organs and non-specific drug release following systemic administration often lead to significant side effects. Thus, strategies that can exacerbate tumor hypoxia to intensify HAP efficacy while simultaneously avoiding potential side effects remain highly desirable. Notably, AQ4N activation is favored by tumor hypoxia together with a weakly acidic microenvironment rather than a strongly acidic one, so approaches that jointly modulate local O_2_ and pH within a mild range are preferable.

Black phosphorus nanosheets (BPNSs), a nanomaterial of two-dimensional black phosphorus, have recently emerged for different bioapplications due to their distinctive physical and chemical properties, such as good biocompatibility, thickness-dependent band gap, and efficient photothermal performance [[23], [24], [25], [26], [27], [28]]. In particular, BPNSs possess a wrinkled honeycomb-like structure, which increases the specific surface area, and each P atom in BPNSs has a lone pair of electrons. The increased surface area and the lone pairs exposed on the surface together endow BPNSs with a unique chemical activity to react with various substances. For instance, BPNSs are prone to react with O_2_ and water (H_2_O) molecules in the ambient environment [29]. As the reaction progresses, the structure of BPNSs would be gradually destroyed, eventually degrading into phosphates that are generally non-toxic and easily metabolized by biospecies [30,31]. The unique chemical activity, which has been well observed and can potentially mitigate the risk associated with the long-term use of BPNSs in vivo, unfortunately, has not been profoundly explored.

**Fig. 1:**
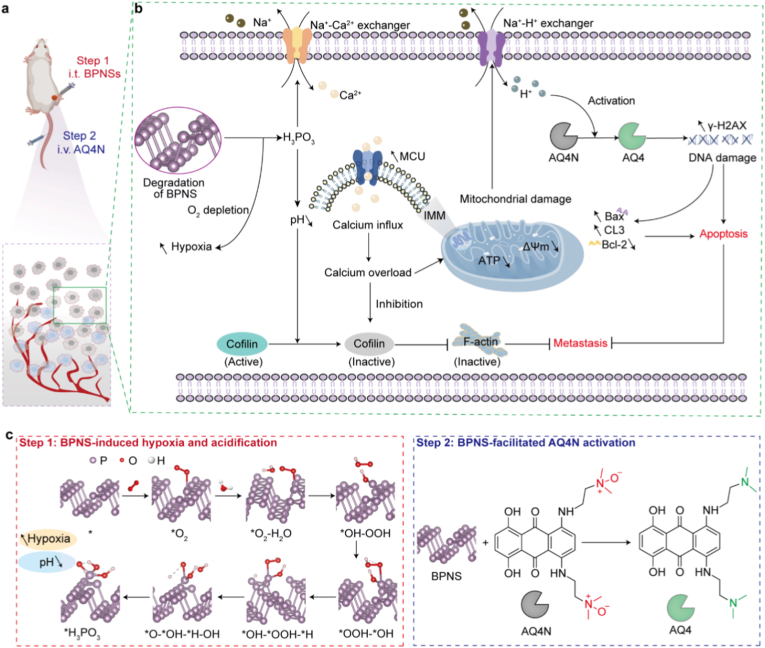
Schematic illustration of the BPNSs + AQ4N strategy for enhanced cancer therapy. (a) Two-step injection protocol: intratumoral (i.t.) injection of BPNSs followed by intravenous (i.v.) injection of AQ4N. (b) BPNS-enhanced anticancer effects in tumor tissues. BPNS degradation consumes O_2_ and generates acid, creating a hypoxic and weakly acidic TME that promotes AQ4N activation. The combined treatment perturbs Ca^2+^ homeostasis, causes mitochondrial damage and apoptosis, induces DNA damage, and inhibits metastasis. (c) Degradation of BPNSs (left) and BPNS-assisted activation of AQ4N (right). CL3, Bcl-2, BAX, MCU, IMM, ATP, ΔΨm, and γ-H2AX denote cleaved caspase-3, B cell lymphoma 2, Bcl-2-associated X protein, mitochondrial calcium uniporter, inner mitochondrial membrane, adenosine triphosphate, mitochondrial membrane potential, and histone H2AX phosphorylation, respectively.

Here, we exploit the distinctive degradation chemistry of BPNSs to develop a strategy for enhancing the anticancer efficacy of HAPs. Unlike most existing nanocarrier-based co-delivery systems, which lack the capacity for in situ TME modulation, or strategies that address only hypoxia or acidosis alone, our approach simultaneously achieves three key functions through a single material: oxygen depletion, tumor acidification, and direct prodrug activation. Furthermore, this strategy leverages the spontaneous degradation of BPNSs without requiring external stimuli, offering greater simplicity and biosafety. Specifically, in our strategy (Fig. 1), BPNSs and AQ4N are sequentially injected based on our findings that BPNSs can facilitate the activation of AQ4N to the highly cytotoxic AQ4 (1,4-bis[[2-(dimethylamino)ethyl]amino]-5,8-dihydroxyanthracene-9,10-dione). Upon intratumoral injection, BPNSs react with O_2_ and H_2_O and degrade within the tumor, thereby aggravating tumor hypoxia and acidifying the surrounding environment. Under the intensified hypoxic and acidic conditions, the subsequently administered AQ4N undergoes efficient conversion to AQ4 with the further assistance of BPNS-AQ4N interactions, ultimately exerting a chemotherapeutic effect. Concurrently, BPNS degradation disrupts the mitochondrial calcium uniporter (MCU) pathway and decreases cofilin activity, leading to Ca^2+^ overload, mitochondrial dysfunction, and inhibition of cell motility. As a result, the combination of BPNSs and AQ4N provides robust anticancer efficacy, effectively inhibiting tumor growth and reducing systemic toxicity.

## 2 Materials and methods

### 2.1 Materials

Tin (Sn) powder and iodine (I_2_) granules were purchased from Aladdin. Red phosphorus and tris(4,7-diphenyl-1,10-phenanthroline) ruthenium (II) dichloride (RDPP, a luminescent oxygen sensor) were obtained from Alfa Aesar. Banoxantrone dihydrochloride (AQ4N) and carboxy-SNARF 1 (5/6-mixture) (SNARF 1, a pH-sensitive fluorescence probe) were provided by MedChemExpress (MCE). N-methyl-2-pyrrolidone (NMP) was obtained from Sigma-Aldrich. 4′,6-diamidino-2-phenylindole dihydrochloride (DAPI), 3-(4,5-dimethyl-2-thiazolyl)-3,5-diphenyl-2H-tetrazol-3-ium bromide (MTT), Hanks' Balanced Salt Solution (HBSS), 5,5′,6,6′-tetrachloro-1,1′,3,3′-tetraethylbenzimidazolylcarbocyanine iodide (JC-1), Fluo4-AM, and MitoTracker dyes were ordered from Beyotime Biotechnology. 2′,7′-bis-(2-carboxyethyl)-5-(and-6)-carboxyfluorescein acetoxymethyl ester (BCECF-AM, a pH-sensitive probe) and fluorophore-conjugated secondary antibodies, including Alexa Fluor™ 488 goat anti-mouse IgG (F2761), Alexa Fluor™ 488 goat anti-rabbit IgG (F2765), Alexa Fluor™ 594 goat anti-mouse IgG (T2769), and Alexa Fluor™ 594 donkey anti-rabbit IgG (PA1-28565), were purchased from Thermo Fisher Scientific. Other materials were obtained from Aladdin, unless otherwise noted. All chemicals were used as received without further purification, unless otherwise noted.

### 2.2 Characterization

Transmission electron microscopy (TEM) images were recorded on a transmission electron microscope (JEM-1400plus, JEOL). High-resolution TEM images and TEM images of cells were obtained using a TECNAI G 20 TWIN (FEI) transmission electron microscope. Hydrodynamic diameter analysis was performed on a Zetasizer Nano instrument (Malvern). pH of all aqueous samples was measured with a calibrated pH meter (S220, Mettler-Toledo). Changes in oxygen levels of aqueous samples were measured using a portable dissolved oxygen meter (ST 300D, OHAUS). Extinction spectra were collected on a UV-vis spectrometer (UV-3600, Shimadzu). Cells were typically observed by an E200 Nikon microscope, and fluorescence images were obtained using an inverted Nikon microscope (IX73 or Ts2). X-ray photoelectron spectroscopy (XPS) results were obtained on a Thermo Fisher instrument (K-Alpha). A BD FACSCanto II flow cytometer (BD Biosciences) was used to analyze cells. Western blot images were acquired using a Tanon imaging system (4800, Tanon). ^1^H nuclear magnetic resonance (NMR) spectra were measured on a JEOL spectrometer (JNM-ECZ400S, 400 MHz). Adenosine triphosphate (ATP) assay results were obtained on a Thermo Fisher instrument (Luminoskan Ascent).

### 2.3 Synthesis of black phosphorus [32]

In a typical experiment, SnI_4_ was first synthesized according to a reported method [33]. 300 mg of red phosphorus, 12 mg of Sn, and 6 mg of SnI_4_ were then thoroughly mixed and sealed in a quartz ampoule under a vacuum pressure (∼–0.1 MPa). The sealed ampoule was horizontally positioned in a muffle furnace and heated to 887 K over 1 h. Subsequently, a controlled cooling process was initiated. The temperature was first decreased to 823 K at a rate of 2 K/min, followed by a slow rate to 773 K at 0.1 K/min before naturally cooling to room temperature. The resulting black phosphorus was washed with hot toluene and alcohol to remove any residual impurities. Finally, the purified black phosphorus was vacuum-dried and stored under a N_2_ atmosphere for further use.

### 2.4 Synthesis of black phosphorus nanosheets

Black phosphorus nanosheets (BPNSs) were prepared via liquid-phase exfoliation [34]. Initially, bulk black phosphorus was mixed with 5 mL of NMP in an agate mortar and ground for 10 min. After that, the dispersion was transferred to a centrifuge tube, and an additional 5 mL of NMP was added to the remaining mixture for further grinding. This process was repeated 10 times. To prevent oxidation, the resulting dispersion was purged with nitrogen gas and sealed with parafilm. Subsequently, the dispersion was ultrasonicated in an ice bath for 12 h and then centrifuged (3000 rpm, 20 min) to collect the supernatant. The obtained supernatant was further centrifuged (16000 rpm, 20 min) to isolate the exfoliated BPNSs. The resulting sediment was collected, thoroughly washed, and dispersed in water for further use.

### 2.5 Observation of the degradation of black phosphorus nanosheets on a copper grid

Generally, 10 μL of an aqueous dispersion of BPNSs (1 mg/mL) was first deposited onto a copper grid and then dried for TEM characterization. After that, the grid was stored in a desiccator with ∼75% relative humidity (using a saturated NaCl solution) at ambient temperature (22–25 °C). The grid was withdrawn from the desiccator on specified days for TEM characterization.

### 2.6 Oxygen consumption by black phosphorus nanosheets

To evaluate the oxygen consumption of BPNSs, two aqueous solutions were prepared. One contained BPNSs, and the other was pure water, serving as a control. Both solutions were prepared with an identical volume and maintained under the same experimental conditions. The dissolved oxygen levels in each solution were monitored over 100 h using a dissolved oxygen meter.

### 2.7 X-ray photoelectron spectroscopy analysis

Two samples were prepared for XPS analysis. Briefly, exfoliated BPNSs dispersed in NMP purged with N_2_ were divided into two parts. One part was treated to disperse the BPNSs in water for preparing the first sample. The BPNSs dispersed in water were dropped onto a silicon wafer and dried under vacuum. Subsequently, the silicon wafer holding the BPNSs was stored at ∼75% relative humidity (saturated NaCl solution) and ambient temperature (22–25 °C), with regular daily light exposure (∼12 h). After 14 days, the sample was transferred to a dry (sealed) amber glass vial purged with N_2_ prior to analysis. The other portion of the NMP dispersion was stored in the dark for 14 days, after which it was treated to disperse the BPNSs in water for preparing the second sample as a control. After the BPNSs on the silicon wafer were dried under vacuum, the silicon wafer was transferred directly to a dry (sealed) amber glass vial purged with N_2_ prior to analysis.

### 2.8 X-ray diffraction analysis

Two samples were prepared for XRD analysis. First, the freshly prepared BPNSs dispersed in water were dried directly for analysis. Next, the aqueous dispersion of the BPNSs was placed under ambient conditions, after which it was dried for further analysis.

### 2.9 Mass spectrometry analysis

To investigate the changes in AQ4N in the presence and absence of BPNSs, a mixture of AQ4N (200 μL, 2 mg/mL) and BPNSs (200 μL, 2 mg/mL) was incubated for 24 h at a certain pH (pH = 7.4 and 5.5, Tris-HCl buffer, 20 mM). The mixture was then centrifuged (6000 rpm, 10 min) to collect the supernatant. Finally, the resulting supernatant was analyzed using a matrix-assisted laser desorption ionization–time of flight (MALDI-TOF) mass spectrometer (4800, AB Sciex).

### 2.10 General cell culture and treatment

The 4T1 cell line (a mouse breast cancer cell line) was cultured in Roswell Park Memorial Institute (RPMI)-1640 medium supplemented with 10% fetal bovine serum (FBS, ST30-3302, PAN) at 37 °C in a humidified atmosphere containing 5% CO_2_. The MCF-7 (a human breast cancer cell line) and HeLa (a human cervical cancer cell line) cell lines were cultured in Dulbecco's Modified Eagle Medium (DMEM) supplemented with 10% FBS under the same conditions as the 4T1 cells. To wash the cells, phosphate-buffered saline (PBS, 10 mM) was used, unless otherwise noted.

### 2.11 Animal experiments

Female BALB/c mice (5-6 weeks old) were provided by the Comparative Medicine Centre of Yangzhou University. All animal experiments were ethically approved (ethical permit number: No. 20241113429699) and conducted under the guidance of the Zhejiang Provincial People's Hospital, Affiliated People's Hospital, and Hangzhou Medical College in accordance with relevant laws and institutional guidelines.

### 2.12 Theoretical calculation

The structural and electronic properties of BPNSs-M∗(M = H_2_O, O_2_ and AQ4N) were calculated using the Vienna Ab-initio Simulation Package (VASP) program based on density functional theory within the generalized gradient approximation with the exchange correlation functional of Perdew-Burke-Ernzerhof. The cut-off energy was set to 400 eV. The structures were relaxed until the atomic forces were less than 0.01 eV/Å per atom and total energies are converged to 10^−6^ eV. The Brillouin zones were sampled with a grid of 1 × 1 × 1 mesh points according to the Monkhorst-Pack procedure for BPNSs-M∗(M = H_2_O, O_2_ and AQ4N). The model was built on the surface layer of a 4 × 4 black phosphorus supercell containing 64 atoms (lattice parameter: *a* = 18.20970 Å, *b* = 13.22440 Å, α = β = γ = 90°) for calculating of energy. For the molecular dynamic simulations, we used a 5 × 7 black phosphorus supercell containing 140 atoms (lattice parameter: *a* = 23.23230 Å, *b* = 32.99780 Å, α = β = γ = 90°), as AQ4N was a larger molecule.

## 3 Results and discussion

### 3.1 Preparation and degradation of black phosphorus nanosheets

**Fig. 2:**
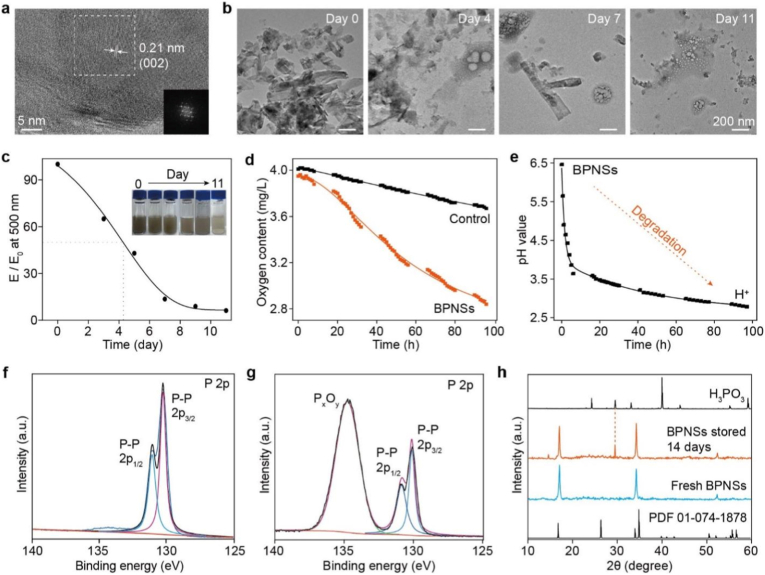
Characterization and degradation of BPNSs. (a) High-resolution TEM image of a BPNS. Inset: fast Fourier transform image of the area indicated by the dashed rectangle. (b) TEM images of BPNSs on days 0, 4, 7, and 11 after ambient exposure. (c) Extinction changes of a non-deoxygenated BPNS dispersion at 500 nm over 11 days. Insets: corresponding photographs. (d) Dissolved oxygen concentration of non-deoxygenated water and a non-deoxygenated aqueous dispersion of BPNSs over 100 h. (e) pH of a non-deoxygenated aqueous dispersion of BPNSs over 100 h. (f, g) High-resolution XPS spectra of P 2p for BPNSs stored (f) in the absence of O_2_, H_2_O, and light and (g) under ambient conditions. (h) XRD patterns of freshly prepared BPNSs and BPNSs dispersed in non-deoxygenated H_2_O. Reference patterns of pure H_3_PO_3_ (top) and black phosphorus (bottom, standard reference) are shown.

We synthesized BPNSs via liquid-phase ultrasonic exfoliation (Fig. S1) [34]. According to the transmission electron microscopy (TEM) characterization, the as-prepared BPNSs are thin with a wide size distribution (Fig. S2). After the BPNSs were dispersed in ethanol, the average hydrodynamic size of BPNSs was ∼220 nm (Fig. S3). High-resolution TEM images of the BPNSs showed that the BPNSs have lattice spacings of 0.21 and 0.33 nm (Fig. 2a and S4). The observed lattice spacings can correspond to the (002) and (021) planes of orthorhombic black phosphorus, respectively, indicating a high crystallinity of the BPNSs.

Subsequently, we monitored the degradation of the obtained BPNSs. The degradation of BPNSs was first studied at ∼75% relative humidity and ambient temperature as BPNSs was found to decompose in the presence of both H_2_O and O_2_ [35]. As shown in Fig. 2b, freshly exfoliated BPNSs loaded on a TEM grid appeared as nanosheets with well-defined edges. After 4 days, protrusions (“bubbles”) were observed on the surface of these BPNSs, which should be due to the degradation of BPNSs. The amount of the bubbles increased in proportion to the duration of the BPNSs’ exposure. By day 11, nearly all BPNSs had degraded, revealing the instability and degradation of BPNSs in the presence of both H_2_O and O_2_. Similarly, BPNSs dispersed in non-deoxygenated H_2_O underwent degradation over time, as indicated by both a colour fading of the BPNS dispersion and a continuous decline in the extinction of the BPNS dispersion at 500 nm (Fig. 2c). According to the extinction spectra (Fig. 2c), the BPNSs were half degraded in H_2_O after ∼4 days. Collectively, the change in extinction of the BPNS dispersion is generally consistent with the morphological changes of BPNSs exposed to ambient conditions, confirming the degradation of BPNSs.

To gain deeper insights into the degradation of BPNSs, the O_2_ consumption during the degradation of BPNSs in H_2_O was evaluated. We monitored the dissolved O_2_ concentration in a sealed aqueous dispersion of BPNSs without deoxygenation (Fig. S5). Dramatic O_2_ depletion was observed, with the concentration of dissolved oxygen decreasing from ∼4.0 to 2.8 μg/mL, which was ∼4-fold greater than that of pure H_2_O (Fig. 2d). Meanwhile, the pH of the BPNS dispersion changed progressively from ∼6.5 to 2.7 within 100 h (Fig. 2e). Accordingly, these results demonstrate that BPNSs are first oxidized to phosphorus oxide species (P_x_O_y_) and then react with H_2_O to form phosphorous acid, yielding a decreased pH value. To check the speculation, BPNSs stored under different conditions for 14 days were studied by X-ray photoelectron spectroscopy (XPS). BPNSs stored in the absence of O_2_, H_2_O, and light exhibited exclusive P-P bonding (2p1/2: ∼131 eV, 2p3/2: ∼130 eV, Fig. 2f), while the BPNSs exposed under ambient conditions (75% relative humidity) developed a new P-O bond peak at ∼135 eV (Fig. 2g), revealing the formation of phosphorus oxides. Parallelly, the X-ray diffraction (XRD) pattern of the BPNSs dispersed in non-deoxygenated H_2_O was recorded and compared with that of freshly prepared BPNSs. A new diffraction peak at ∼29° that can be attributed to H_3_PO_3_ was observed (Fig. 2h), which supports our assumption. Together, these results indicate a coherent degradation pathway of BPNSs, in which O_2_ drives the oxidation of BPNSs and H_2_O further reacts with the generated phosphorus oxides to phosphorous acid.

### 3.2 Prodrug activation by black phosphorus nanosheets

**Fig. 3:**
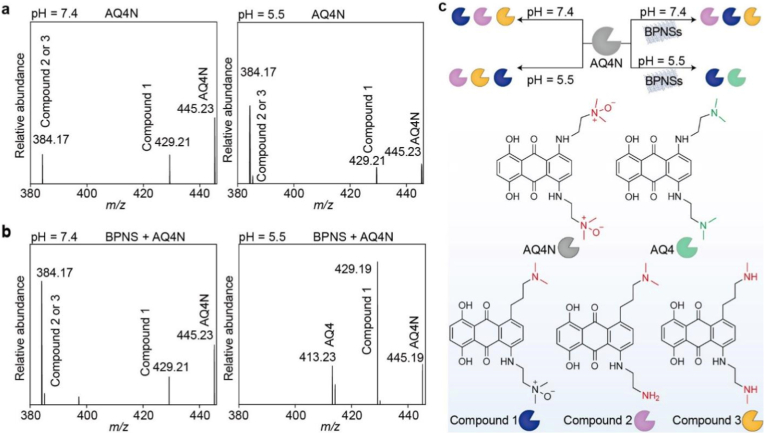
Activation of AQ4N by BPNSs. (a, b) Mass spectra of AQ4N incubated in the absence and presence of BPNSs under (a) slightly alkaline (pH = 7.4) and (b) acidic (pH = 5.5) conditions for 24 h. (c) Schematic illustration of AQ4N evolution in the absence and presence of BPNSs under slightly alkaline and acidic conditions.

To evaluate whether BPNSs can promote the activation of the hypoxia-responsive prodrug AQ4N (Figs. S6 and S7), we incubated AQ4N with and without BPNSs in Tris buffer at pH 7.4 and 5.5 for 24 h and analyzed the mixtures by mass spectrometry (Fig. 3a and b). Under slightly alkaline conditions (pH = 7.4, the typical physiological pH), the mass spectra confirmed the presence of AQ4N (*m*/*z* 445) and AQ4N derivatives (Compounds 1 and 2/3, Fig. 3c), but not the cytotoxic metabolite AQ4, regardless of the presence or absence of BPNSs. Similar results were observed when AQ4N alone was incubated under acidic conditions (pH = 5.5). In stark contrast, the conversion of AQ4N to AQ4 occurred in the presence of BPNSs under acidic conditions, as evidenced by the detection of both Compound 1 and AQ4 (*m*/*z* 413) in the mass spectrum (Fig. 3b). These mass spectrometry data provide direct experimental evidence that BPNSs can selectively promote AQ4N-to-AQ4 conversion under acidic conditions (Fig. 3c). Notably, the incubated mixtures were not deoxygenated; thus BPNS degradation could occur concurrently, creating a low-oxygen and acidic local environment that further favors AQ4N activation.

**Fig. 4:**
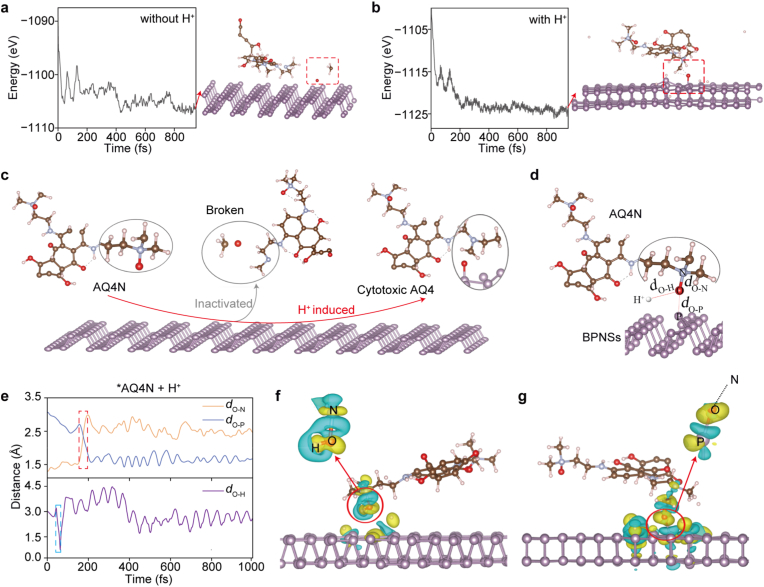
Theoretical simulation of the AQ4N activation by BPNSs. (a, b) Molecular dynamics assessment of AQ4N states in the presence of BPNSs under nonacidic and acidic conditions, respectively. (c) Schematic illustration of AQ4N activation by BPNSs. (d) Illustration of the monitored three interatomic distances (*d*_O-N_, *d*_O-P_, and *d*_O-H_). (e) Evolution of the monitored distances over time during the activation of AQ4N. (f, g) Electron density difference during (f) the transient formation of protonation and (g) the O-N bond cleavage and concurrent O-P bond formation, respectively.

To understand the role of BPNSs in the activation of AQ4N, we employed molecular dynamics simulations to study AQ4N under different conditions by assuming that BPNSs may participate in AQ4N activation. We initiated the simulation by positioning an AQ4N molecule on the surface of a BPNS under both nonacidic and acidic conditions without considering other factors (Fig. 4a and b). Upon the simulation reaching dynamic equilibrium under nonacidic conditions, the AQ4N molecule underwent a structural distortion, characterized by the cleavage of a methyl group and an oxygen atom (Fig. 4a, dashed box), inhibiting the activation of AQ4N. Differently, under acidic conditions (i.e., in the presence of H^+^), selective detachment of an oxygen atom from the AQ4N molecule occurred for AQ4N activation, followed by the migration and adsorption of the oxygen atom onto the BPNS (Fig. 4b, dashed box). These simulation results, consistent with the experimental results, suggest that BPNSs can activate AQ4N under acidic conditions, in which H^+^ should play a critical role. According to these theoretical findings and our experimental observations, we propose a mechanistic model of the selective activation of AQ4N by BPNSs under acidic conditions (Fig. 4c).

To gain deeper insights into the role of H^+^ in BPNS-induced activation of AQ4N, we analyzed the local bonding dynamics and charge redistribution during the activation (Fig. 4d–g). Specifically, we monitored three key interatomic distances illustrated in Fig. 4d, the distances of O-N (*d*_O-N_), O-P (*d*_O-P_), and O-H (*d*_O-H_), to track dynamic changes in bonding states throughout the activation. Our calculated results show that the desorption of an oxygen atom from AQ4N is consistently accompanied by a gradual increase in *d*_O-N_ and a concurrent decrease in *d*_O-P_ (Fig. 4e, highlighted by a red dashed box), indicating the cleavage of the O-N bond and concurrent formation of the O-P bond. Importantly, the calculated results reveal that the transition of *d*_O-N_ and *d*_O-P_ is preceded by a significant decrease in *d*_O-H_ (Fig. 4e, highlighted by a blue dashed box), suggesting the transient formation of an O-H bond due to proton engagement. The coupled behavior of *d*_O-N_, *d*_O-P_, and *d*_O-H_ indicates a two-step activation sequence initiated by H^+^ binding and culminating in O-N bond rupture and O-P bond formation. To validate this two-step activation, we further analyzed the differential charge density during the H^+^-initiated activation of AQ4N (Fig. 4f and g). The results show a significant electron accumulation around the N-bound O atom upon protonation. Such H^+^-caused electron redistribution can weaken the O-N bond but enhance the affinity of O toward P, facilitating the formation of the O-P bond and cytotoxic AQ4. Briefly, these simulated results reveal a H^+^-triggered activation of AQ4N by BPNSs. Taking the degradation of BPNSs, the BPNS-based activation of AQ4N, and the acidic and hypoxic tumor microenvironment together, we deduce that BPNSs can aggravate tumor hypoxia, further acidify tumors, and specifically activate AQ4N in tumors for therapy. Thus, the combination of AQ4N and BPNSs could potentially be an effective and specific cancer treatment.

### 3.3 In vitro evaluation

**Fig. 5:**
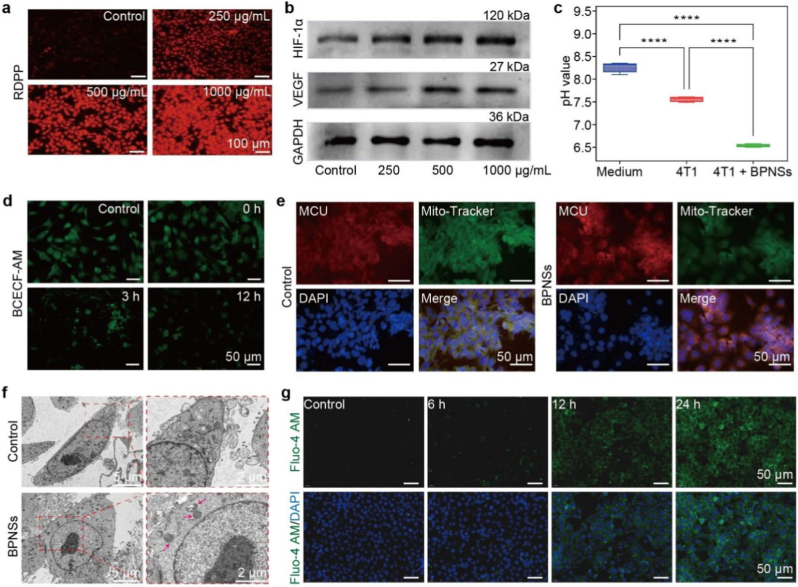
Influence of BPNSs on 4T1 cells. (a) Fluorescence images showing intracellular O_2_ levels (RDPP probe) in 4T1 cells treated with different concentrations of BPNSs. (b) Western blot analysis of HIF-1α, VEGF, and GAPDH expression in 4T1 cells treated with different concentrations of BPNSs. (c) pH values of pure medium and culture media of 4T1 cells treated with or without BPNSs (mean ± SD, n = 3). Statistical significance was analyzed by two-tailed Student's t-test, ∗∗∗∗*P* < 0.0001. (d) Fluorescence images showing intracellular pH changes (BCECF-AM) in 4T1 cells incubated with or without (control) BPNSs over time. (e) Immunofluorescence images of MCU in 4T1 cells treated with or without (control) BPNSs. MCU (red), mitochondria (MitoTracker, green), nuclei (DAPI, blue). (f) TEM images of 4T1 cells incubated with or without (control) BPNSs. Red arrows indicate mitochondrial damage. (g) Fluorescence images showing intracellular Ca^2+^ levels (Fluo4-AM, green) in 4T1 cells treated with or without (control) BPNSs. Nuclei were stained with DAPI (blue). (For interpretation of the references to colour in this figure legend, the reader is referred to the Web version of this article.)

Motivated by the BPNS-involved activation of AQ4N, we investigated the influence of BPNSs on the therapeutic effects of AQ4N in cancer cells to verify our deduction, with initial attention on BPNS-induced O_2_ depletion and acid generation. The intracellular O_2_ consumption of BPNSs was first assessed using an O_2_-sensitive probe, tris(4,7-diphenyl-1,10-phenanthroline) ruthenium (II) dichloride (RDPP), in 4T1 cells (Fig. 5a). In the absence of BPNSs, 4T1 cells exhibited very weak red fluorescence, indicating a high concentration of O_2_, since the red fluorescence intensity of RDPP is inversely correlated with O_2_ concentration. In stark contrast, after incubation with BPNSs, the cells exhibited increased red fluorescence dependent on the concentration of BPNSs (Fig. 5a). Similar results were also observed in other cancer cell lines, such as MCF-7 and HeLa cells (Fig. S8). Further western blot analyses of the 4T1 cells showed that the expression of HIF-1α and its downstream target, vascular endothelial growth factor (VEGF), increased in a BPNS dose-dependent manner (Fig. 5b). These results confirm the efficient intracellular oxygen depletion by BPNSs, resulting in a hypoxic microenvironment at the cellular level.

After 4T1 cells were incubated with BPNSs, the pH value of the cell culture medium was measured, and a significant acidification was observed (Fig. 5c), consistent with the formation of H_3_PO_3_ during oxygen consumption by BPNSs. Intracellular pH was then monitored using a pH-sensitive dye (2′,7′-bis- (2-carboxyethyl)-5-(and-6)-carboxyfluorescein acetoxymethyl ester (BCECF-AM)), which could give decreased green fluorescence with the decline of pH. As shown in Fig. 5d and S9, the intracellular pH of 4T1 cells decreased gradually after the cells were incubated with BPNSs for 0 to 12 h.

Furthermore, fluorescence images and TEM of BPNS-treated 4T1 cells reveal both mitochondrial damage and overexpression of the mitochondrial calcium uniporter (MCU) (Fig. 5e and f), highlighting the anti-tumor effects of BPNSs. As MCU functions as the primary channel for mitochondrial Ca^2+^ influx, its upregulation can facilitate excessive Ca^2+^ entry into mitochondria. Together with the elevated intracellular Ca^2+^ levels revealed by the fluorescence assays (Fig. 5g), we reason that BPNSs can cause mitochondrial damage and mitochondrial Ca^2+^ overload, thereby exhibiting antitumor ability. Notably, the observed intracellular Ca^2+^ overload, together with the decline in pH, likely activates the Na^+^/H^+^ exchanger, which extrudes H^+^ at the expense of Na^+^ influx, leading to an intracellular Na^+^ accumulation [36]. In addition, we observed inhibition of Na^+^/K^+^ adenosine triphosphatase after BPNSs treatment (Fig. S10), which can also increase intracellular Na^+^. Excess Na^+^ can subsequently impair the Na^+^/Ca^2+^ exchanger, further promoting intracellular Ca^2+^ accumulation and mitochondrial Ca^2+^ overload.

**Fig. 6:**
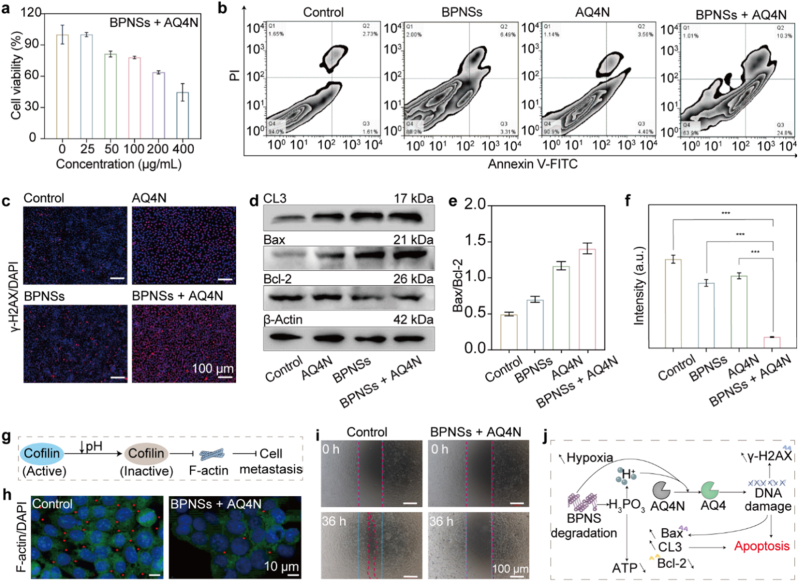
Anticancer cell effect. (a) Viability of 4T1 cells treated with various concentrations of BPNSs + AQ4N (mean ± SD, n = 3). (b) Flow cytometry analyses of 4T1 cells after four different treatments. (c) Fluorescence images of 4T1 cells stained with a γ-H2AX probe in four treatment groups. Cell nuclei were stained with DAPI. (d) Expression of CL3, Bax, Bcl-2, and β-Actin in cells after four different treatments. (e) Quantification of the Bax/Bcl-2 ratio (mean ± SD, n = 3). (f) Measured ATP levels in cells treated with four treatment formulations (mean ± SD, n = 4). Statistical significance was analyzed by one-way ANOVA followed by Tukey's multiple-comparison test, ∗∗∗*P* < 0.001. (g) Schematic illustration of the pH-induced metastasis inhibition. (h) Fluorescence images showing F-actin expression (green) in the control and the BPNSs + AQ4N-treated groups. Cell nuclei were stained with DAPI. (i) Wound-healing assay of untreated (control) and BPNSs + AQ4N-treated cells. Blue and red dashed lines indicate the edge of the gap before and after different treatments. (j) Schematic illustration of the BPNSs + AQ4N therapy in cells. (For interpretation of the references to colour in this figure legend, the reader is referred to the Web version of this article.)

Having established BPNS-induced TME modulation (O_2_ depletion and acidification) and its downstream biological effects on Ca^2+^ homeostasis and mitochondrial function, we next assessed the therapeutic effects brought by the combination of AQ4N and BPNSs in vitro by studying the in vitro antitumor effects of four treatment formulations using 4T1 cells, including no treatment (control), applying AQ4N alone, applying BPNSs alone, and applying both BPNSs and AQ4N (BPNSs + AQ4N). The results of the 3-(4,5-dimethylthiazol-2-yl)-2,5-diphenyltetrazolium bromide (MTT) assay show that over 75% and 65% of the 4T1 cells preserved their viability in the presence of AQ4N or BPNSs alone, respectively, even at a high concentration of 400 μg/mL (Fig. S11). In contrast, co-treatment with BPNSs and AQ4N eliminated over 50% of the 4T1 cells (Fig. 6a), demonstrating a synergistic lethality. Flow cytometry analyses reveal that the combination of BPNSs and AQ4N induces the highest level of cell apoptosis (35.09%) as compared to the control (4.34%), AQ4N alone (7.96%), and BPNSs alone (9.80%) treatments (Fig. 6b), further confirming the synergistic antitumor effects of BPNSs and AQ4N.

To identify the antitumor mechanism, we first used 5,5′,6,6′-tetrachloro-1,1′,3,3′-tetraethylbenzimi dazolylcarbocyanine iodide (JC-1) to evaluate the mitochondrial membrane potential (MMP) of cells after different treatments. As shown in Fig. S12, only the BPNSs + AQ4N-treated cells exhibited dominant green fluorescence, indicating severe MMP collapse. Subsequently, DNA damage was assessed via the histone H2AX phosphorylation (γ-H2AX) staining assay, in which observed fluorescent foci could reveal DNA double-strand breaks. The BPNSs + AQ4N-treated cells showed a substantial fluorescence increase in γ-H2AX staining when compared with other treated groups (Fig. 6c), indicating a severe genotoxic stress and extensive DNA fragmentation after the combined treatment. Meanwhile, western blot analysis was performed to evaluate the expression of the B cell lymphoma 2 (Bcl-2) family proteins, which are key regulators of the mitochondrial pathway involved in cell death. The observed upregulation of Bcl-2-associated X protein (Bax) and downregulation of Bcl-2 (Fig. 6d and e), together with increased cleaved caspase-3 (CL3) expression, corroborate the flow cytometry results, showing that the BPNSs + AQ4N-treated cells exhibited enhanced apoptosis. Furthermore, we observed an ∼85% decrease in adenosine triphosphate (ATP) in the BPNSs + AQ4N-treated cells and much higher ATP levels in other treated groups (Fig. 6f and S13). This indicates a hypoxia-triggered starvation therapy caused by the combination of AQ4N and BPNSs. These in vitro results reveal that the combination of AQ4N and BPNSs can trigger multiple antitumor therapies and synergistically enhance the therapeutic effects of AQ4N. Collectively, the TEM-visualized mitochondrial structural damage, elevated intracellular Ca^2+^, MCU upregulation, MMP collapse, Annexin V/PI flow cytometry, and the Bax/Bcl-2 and CL3 changes form a coherent evidence chain for BPNS-triggered mitochondrial dysfunction and apoptosis.

In addition, considering that BPNSs can acidify the intracellular environment, and cofilin, a pH-sensitive actin-regulatory protein, would undergo functional inactivation and disrupted actin dynamics under acidic conditions (Fig. 6g), we studied the effects of the BPNSs + AQ4N treatment on F-actin and cell metastasis. Fluorescence assays reveal that the F-actin cytoskeletons of cells in the BPNSs + AQ4N-treated group became unclear (Fig. 6h), indicating a decreased cofilin activity. Subsequent wound-healing assays showed that untreated cells (the control group) achieved ∼77% closure after 36 h, while the BPNSs + AQ4N-treated group achieved only 0.4% closure (Fig. 6i and S14), clearly revealing the inhibition of cell motility by the BPNSs + AQ4N treatment. Collectively, the aforementioned in vitro results suggest that the combination of AQ4N and BPNSs can deplete intracellular oxygen, acidify the intracellular environment, and synergistically potentiate the therapeutic effects of AQ4N (Fig. 6j).

### 3.4 Antitumor therapy

**Fig. 7:**
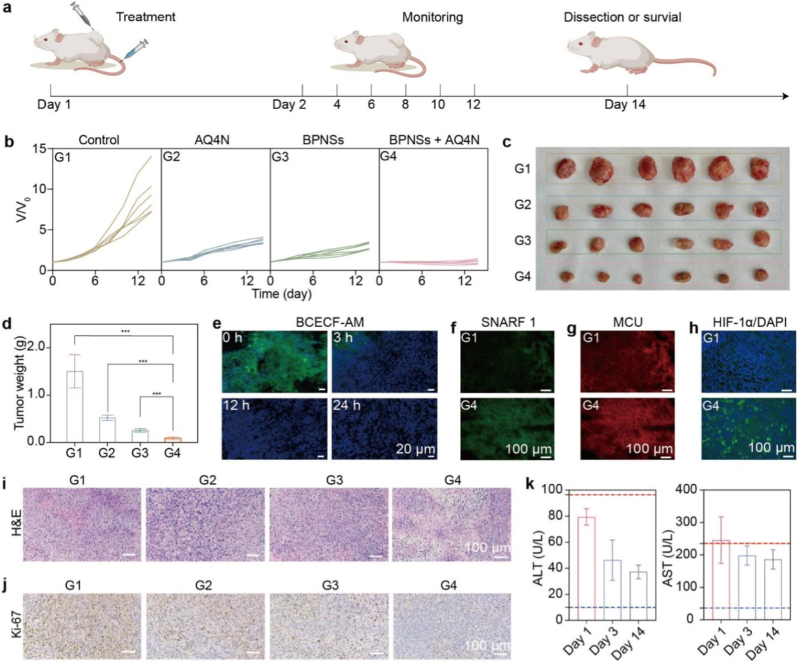
In vivo antitumor efficacy. (a) Schematic of the treatment protocol. (b) Tumor growth curves of mice in the four treatment groups (n = 6 per group). G1: PBS control (i.v.); G2: AQ4N alone (i.v., 4 mg/kg); G3: BPNSs alone (i.t., 4 mg/kg); G4: BPNSs + AQ4N (i.t. 4 mg/kg BPNSs, i.v. 4 mg/kg AQ4N). (c) Photographs of excised tumors on day 14. (d) Average tumor weights on day 14 (mean ± SD, n = 6). ∗∗∗*P* < 0.001. (e) Fluorescence images of tumor sections stained with BCECF-AM at 0, 3, 12, and 24 h after BPNSs injection. Nuclei: DAPI (blue). (f, g) Fluorescence images of tumor sections from G1 (control) and G4 (BPNSs + AQ4N) groups showing (f) intratumoral pH (SNARF 1) and (g) MCU levels (MCU antibody, red) in the same tumor region. (h) HIF-1α immunofluorescence in tumor sections from G1 and G4 groups. Nuclei: DAPI (blue). (i) H&E staining of tumor sections from all four treatment groups. (j) Ki-67 immunohistochemistry of tumor sections from all four groups. (k) Serum ALT and AST levels in BPNSs + AQ4N-treated mice on days 1, 3, and 14. Blue and red dashed lines indicate the lower and upper limits of the normal reference range, respectively. (For interpretation of the references to colour in this figure legend, the reader is referred to the Web version of this article.)

According to the in vitro results, we designed a two-step delivery strategy, which involved an intratumoral (i.t.) injection of BPNSs and a subsequent intravenous (i.v.) injection of AQ4N, to evaluate the therapeutic effects of combining BPNSs and AQ4N in vivo. It should be mentioned that the two-step delivery strategy was designed to minimize the undesired activation and side effects of AQ4N. The sequential two-step injection ensures that BPNS-mediated TME modulation occurs first, creating favorable local conditions before AQ4N administration. AQ4N is a small-molecule hypoxia-activated prodrug that has been previously demonstrated to be systemically administrable and capable of reaching tumor tissues after intravenous injection through tumor vascular extravasation and interstitial diffusion [21,22]. In our in vivo experiments, 4T1 tumor-bearing mice were randomly divided into four groups: (G1) a control group (i.v. injection, phosphate buffered saline (PBS)), (G2) an AQ4N monotherapy (i.v. injection, 4 mg/kg) group, (G3) a BPNSs monotherapy (i.t. injection, 4 mg/kg) group, and (G4) a BPNSs + AQ4N combined therapy (i.t. injection of 4 mg/kg BPNSs, i.v. injection of 4 mg/kg AQ4N) group. The mice were then monitored at different times over 14 days, as illustrated in Fig. 7a.

Over the 14 days, the body weight of mice in each group was very similar despite different treatments (Fig. S15). Nevertheless, tumors in the control group exhibited progressive growth (Fig. 7b–d). Tumors in mice treated by AQ4N alone showed moderate growth suppression (Fig. 7b–d), which should be due to limited AQ4N activation. Similarly, tumors in mice treated by BPNSs alone had a comparable growth suppression due to the antitumor ability of BPNSs (Fig. 7b–d). In stark contrast, BPNSs + AQ4N-treated tumors were dramatically inhibited with a rate of ∼90% (Fig. 7b–d), suggesting a good antitumor effect of the combination of BPNSs and AQ4N.

To obtain a deeper understanding of the antitumor effects in vivo, we first studied the tumor pH after the injection of BPNSs using BCECF-AM. Similar to the results observed in cells, the treated tumor became more acidic over time after the injection of BPNSs (Fig. 7e). In addition, tumor in the BPNSs + AQ4N-treated groups showed a much lower pH, as revealed by the increased green fluorescence of the Carboxy-SNARF 1 (5/6-mixture) (SNARF 1) probe (Fig. 7f and S16), compared to those in the control group. Due to the acidic environment caused by BPNSs, elevated MCU expression in tumors was observed in the BPNSs + AQ4N-treated group (Fig. 7g), suggesting mitochondrial Ca^2+^ overload. Further ex vivo fluorescence staining assays showed increased HIF-1α expression in tumors of the BPNSs + AQ4N-treated group (Fig. 7h and S16), indicating enhanced tumor hypoxia caused by BPNSs. These results are similar to those observed in vitro and suggest the important role of BPNSs.

In the following set of experiments, hematoxylin and eosin (H&E) staining was first applied and confirmed the effective tumor cell killing by the BPNSs + AQ4N treatment (Fig. 7i). Ki-67 immunohistochemistry analysis revealed reduced tumor cell proliferation in the BPNSs + AQ4N-treated group. Cells in the proliferative cycle, identified by dark brown 3,3′-diaminobenzidine staining, were significantly fewer in the BPNSs + AQ4N-treated group than those in other groups (Fig. 7j). Meanwhile, blood biochemistry assays and complete blood counts remained normal post-treatment (Fig. 7k–S17, and S18). Notably, the BPNSs + AQ4N treatment normalized the elevated white blood cell count as compared to the control treatment (Fig. S18), which may be associated with reduced tumor-associated inflammatory burden, although further immunological studies are needed. Additionally, H&E staining confirmed the absence of noticeable damage to major organs (Fig. S19). Taken together, these results demonstrate that the BPNSs + AQ4N treatment is an effective antitumor strategy with preliminary short-term biosafety within the treatment period: no obvious body weight loss, abnormal blood biochemical/hematological changes, or apparent histological damage to major organs was observed, highlighting its potential for further preclinical development.

## 4 Conclusion

In summary, we have demonstrated that the intrinsic degradation chemistry of BPNSs can be harnessed to potentiate hypoxia-activated prodrug therapy through three interconnected functions: oxygen depletion, local acidification, and direct assistance of AQ4N-to-AQ4 conversion. Mass spectrometry and molecular dynamics simulations established that BPNSs facilitate proton-assisted O-N bond cleavage in AQ4N selectively under acidic conditions, while in vitro and in vivo experiments confirmed that BPNS-mediated TME modulation leads to mitochondrial Ca^2+^ overload, extensive DNA damage, and inhibition of cell motility, collectively achieving effective tumor growth inhibition in a 4T1 mouse model. Preliminary biosafety assessment revealed no apparent acute toxicity or major organ damage. This work establishes a strategy that exploits nanomaterial degradation as a functional element rather than treating it as a stability concern, and we anticipate that this principle will inform the design of other degradation-assisted therapeutic platforms.

## Ethics statement

All animal experiments were ethically approved (ethical permit number: No. 20241113429699) and conducted under the guidance of the Zhejiang Provincial People's Hospital, Affiliated People's Hospital, and Hangzhou Medical College in accordance with relevant laws and institutional guidelines.

## Funding

The work was supported by 10.13039/501100001809National Natural Science Foundation of China (62405081).

## CRediT authorship contribution statement

**Nan Yang:** Formal analysis, Investigation, Validation, Visualization, Writing – original draft. **Dabao Zha:** Validation, Visualization, Writing – original draft. **Changyu Cao:** Investigation, Writing – original draft. **Ruigang Liu:** Investigation, Validation. **Hanjun Sun:** Validation. **Xuejiao Song:** Writing – original draft. **Yanling Li:** Methodology, Supervision. **Xiaoji Xie:** Writing – original draft, Writing – review & editing. **Xiaochen Dong:** Project administration, Supervision, Writing – review & editing. **Yu Cai:** Project administration, Supervision.

## Declaration of competing interest

The authors declare that they have no known competing financial interests or personal relationships that could have appeared to influence the work reported in this paper.

## Data Availability

Data will be made available on request.
