# Supplementary material for: Black phosphorus nanosheet-promoted prodrug activation for enhanced cancer therapy
Source: Mater Today Bio. 2026 May 17;38:103244. doi: 10.1016/j.mtbio.2026.103244 (PMC13214534; doi:10.1016/j.mtbio.2026.103244)
Supplement: Multimedia component 1 [file mmc1.docx]

**Supporting Information**

Black Phosphorus Nanosheet-Promoted Prodrug Activation for Enhanced Cancer Therapy

*Nan Yang,^a,b^ Dabao Zha,^c^ Changyu Cao,^b^ Ruigang Liu,^b^ Hanjun Sun,^b^ Xuejiao Song,^b*^ Yanling Li,^c^ Xiaoji Xie,^b*^ Xiaochen Dong^b,c*^, Yu Cai^a*^*

^a^Center for Rehabilitation Medicine, Rehabilitation & Sports Medicine Research Institute of Zhejiang Province, Department of Rehabilitation Medicine, Cancer Center, Zhejiang Provincial People's Hospital (Affiliated People's Hospital), Hangzhou Medical College, Hangzhou 310014, China.

*E-mail: caiyu@hmc.edu.cn*

^b^State Key Laboratory of Flexible Electronics (LoFE) & Institute of Advanced Materials (IAM), School of Flexible Electronics (Future Technologies), Nanjing Tech University (NanjingTech), Nanjing 211816, China

*E-mail:* [*xjsong@njtech.edu.cn*](mailto:xjsong@njtech.edu.cn)*, iamxjxie@njtech.edu.cn*

^c^School of Chemistry & Materials Science, School of Physics and Electronic Engineering, Jiangsu Normal University, Xuzhou 221116, China

*E-mail:* [*iamxcdong@njtech.edu.cn*](mailto:iamxcdong@njtech.edu.cn)

**General Experimental Procedures**

**In vitro experiments**

**Intracellular oxygen content detection**

4T1 cells were seeded in 24-well plates at a density of 2.0 × 10^4^ cells per well and cultured overnight to allow adherence. The cells were then treated with varying concentrations of BPNSs (0 (control), 250, 500, and 1000 µg/mL) in fresh medium in a hypoxia (1% O_2_) culture chamber (MIC-101, Bio-Glord). After 12 h, the cells were washed three times with PBS to remove any uninternalized BPNSs. Subsequently, the cells were incubated with the RDPP (10 μM***)*** probe to stain intracellular oxygen levels. After the staining, the cells were washed twice with PBS, fixed with 4% paraformaldehyde (Wuhan Servicebio Technology Co., Ltd.) for 15 min at room temperature, and washed three times with PBS. Finally, fluorescence images of the cells were recorded.

**Western blot assay**

In a typical experiment, 4T1 cells were seeded in a 6-well plate at a density of 2.0 × 10^5^ cells per well. After overnight incubation, the cells were treated with various concentrations of BPNSs (0 (control), 250, 500, and 1000 µg/mL) for 12 h. The cells were then washed with PBS and lysed on ice using a lysis buffer. The protein concentrations in the lysates were determined by the bicinchoninic acid (BCA) protein assay kit (P0010, Beyotime). Equal amounts of total protein from each lysate were then loaded onto a sodium dodecyl sulfate-polyacrylamide gel (12.5%) for electrophoresis and subsequently transferred to polyvinylidene difluoride (PVDF) membranes (ISEQ00010, Millipore). The membranes were blocked with skim milk (5%) and incubated sequentially with the appropriate primary and secondary antibodies. Finally, the protein bands were detected using a chemiluminescence detection system. For cells treated with different treatment formulations, the procedure of the western blot assay is similar.

The following antibodies were used: vascular endothelial growth factor (VEGF) rabbit polyclonal antibody (AF0312, Beyotime), glyceraldehyde-3-phosphate dehydrogenase (GAPDH) rabbit monoclonal antibody (AF1186, Beyotime), B cell lymphoma 2 (Bcl-2) rabbit polyclonal antibody (AB112, Beyotime), Bcl-2-associated X protein (Bax) mouse monoclonal antibody (AB026, Beyotime), β-Actin mouse monoclonal antibody (AF0003, Beyotime), cleaved caspase-3 rabbit polyclonal antibody (25128-1, Proteintech), anti-hypoxia-inducible factor (HIF)-1α rabbit monoclonal antibody (ab179483, Abcam), horseradish peroxidase (HRP)-conjugated goat anti-rabbit immunoglobulin G (IgG) (H+L) (5A00001-2, Proteintech), and HRP-conjugated goat anti-mouse IgG (H+L) (5A00001-1, Proteintech).

**Monitoring the pH of the culture medium and the intracellular pH**

To monitor the pH of the culture medium, 4T1 cells were seeded in 6-well plates at a density of 2.0 × 10^5^ cells per well. Three groups were prepared: (1) the culture medium alone, (2) 4T1 cells cultured without BPNSs, and (3) 4T1 cells treated with BPNSs (200 μg/mL). All groups were incubated for 12 h, after which the pH of the culture medium in each group was measured.

To assess the intracellular pH of 4T1 cells, 4T1 cells were seeded in 6-well plates at a density of 1.0 × 10^5^ cells per well and cultured overnight. The cells were then divided into four groups: one untreated control group and three experimental groups that were treated with BPNSs (200 μg/mL) for different durations (0, 3, and 12 h). After the treatments, all groups were incubated with BCECF-AM (5 μM) for 20 min at 37 °C. The cell nuclei were then stained with DAPI (1 μg/mL) for 10 min. Finally, intracellular pH was analyzed by recording fluorescence under 488 nm excitation.

**Staining of mitochondria and mitochondrial calcium uniporter**

To assess the mitochondrial calcium uniporter (MCU), 4T1 cells were seeded on 18 mm glass coverslips in 6-well plates and cultured overnight. The cells were then treated with PBS (control) or BPNSs (200 μg/mL) for 12 h. After the treatment, the cells were washed with PBS, the mitochondria were stained with MitoTracker Green (20 nM), and the nuclei were stained with DAPI (1 μg/mL). Subsequently, the cells were fixed with 4% paraformaldehyde for 15 min, permeabilized with 0.1% Triton X-100 for 10 min at room temperature, and blocked with 5% bovine serum albumin (BSA) for 30 min. The cells were then incubated overnight at 4 °C with the MCU rabbit monoclonal antibody (14997, Cell Signaling Technology, CST) and washed with PBS. Finally, the cells were incubated with a fluorophore-conjugated secondary antibody for 1 h at room temperature in the dark. Fluorescence images of the cells were recorded for analysis.

**Preparing cells for transmission electron microscopy analysis**

Briefly, to prepare cell samples, 4T1 cells were first seeded in 10 cm culture dishes (3.0 × 10^6^ cells per dish), cultured for 24 h, and treated with PBS or BPNSs (200 μg/mL) for a further 12 h. The cells were then washed and fixed with glutaraldehyde (2.5%). Subsequently, the cells were gently scraped, collected, and further fixed. The resulting cell pellets were rinsed with phosphate buffer (pH = 7.4, 0.1 M) and fixed with osmium tetroxide (OsO_4_, 1%), followed by further rinses with phosphate buffer (pH = 7.4, 0.1 M). The cell samples were then dehydrated in graded ethanol (30-100%), infiltrated, and embedded in resin. Ultrathin sections (80-100 nm) of the embedded cells were obtained using an ultramicrotome (Leica UC7, Leica Microsystems). These sections were collected on copper grids and stained with uranyl acetate and lead citrate to enhance the contrast. After the stained sections were air-dried overnight, they were examined using a transmission electron microscope.

**Intracellular calcium detection**

To monitor intracellular calcium, 4T1 cells were first seeded on 18 × 18 mm coverslips in 6-well plates and cultured overnight. The cells were then treated with BPNSs (200 μg/mL) for various durations (0 (control), 6, 12, and 24 h). At each time point, the cells were washed with HBSS and incubated with 5 μM Fluo-4 AM (Beyotime) for 30 min at 37 °C in the dark. After the staining, the cells were washed twice with HBSS and incubated in fresh HBSS for an additional 10 min to allow complete de-esterification. The cell nuclei were then stained with DAPI (1 μg/mL). Finally, fluorescence images were recorded.

**Na^+^/K^+^** **adenosine triphosphatase activity assay**

Briefly, 4T1 cells were seeded in 6-well plates at a density of 4.0 × 10^5^ cells per well and cultured overnight. The cells were then treated with PBS (control) or BPNSs (200 μg/mL) for 12 h. After the treatment, the cells were washed with PBS and collected. The collected cells were resuspended in 1 mL of Reagent I from the Na^+^/K^+^-adenosine triphosphatase assay kit (Solarbio, BC0065) and lysed by ultrasonication on ice (200 W, 3 s on/10 s off, 30 cycles). The resulting lysate was centrifuged (8000 g, 10 min) at 4 °C. The supernatant was collected and analyzed according to the manufacturer’s protocol. In short, the reaction system was assembled by mixing the supernatant with Reagents I–IV and water (total volume: 200 μL), followed by incubation at 37°C for 30 min. After the addition of the chromogenic and termination reagents, the samples were incubated at 40 °C for 10 min. Finally, the absorbance of the samples at 660 nm was measured using a microplate reader, and the enzyme activity was calculated.

**Cell viability assay**

To evaluate cell viability, 4T1 cells were seeded in 96-well plates at a density of 2.0 × 10^4^ cells per well and incubated overnight. The cells were then divided into three treatment groups: (1) BPNSs alone at concentrations of 0, 50, 100, 200, and 400 μg/mL, (2) AQ4N alone at concentrations of 0, 50, 100, 200, and 400 μg/mL, and (3) a combination of BPNSs and AQ4N (BPNSs+AQ4N) at concentrations of 0, 25, 50, 100, 200, and 400 μg/mL for each component. All treatments were applied for 12 h. After the incubation, 20 μL of MTT solution (5 mg/mL) was added to each well, and the plate was incubated for another 4 h at 37 °C. After removing the supernatant, 200 μL of dimethyl sulfoxide was added to each well to dissolve the formazan crystals. Finally, the absorbance of the resulting solution in each well was measured at 490 nm using a microplate reader (MK3, Thermo Scientific) to determine cell viability.

**Cell apoptosis assay**

Typically, 4T1 cells were seeded in 6-well plates at a density of 4.0 × 10^5^ cells per well and cultured overnight. The cells were then treated for 12 h with four treatment formulations: (1) PBS (control), (2) AQ4N (200 μg/mL), (3) BPNSs (200 μg/mL), and (4) a combination of BPNSs (200 μg/mL) and AQ4N (200 μg/mL).

After the treatment, both the adherent and floating cells were collected by trypsinization and gentle centrifugation (1000 rpm, 5 min). The resulting cell pellets were washed twice with PBS and resuspended in 100 µL of the binding buffer in the Annexin V-fluorescein isothiocyanate/propidium iodide (Annexin V-FITC/PI) apoptosis detection kit (C1062, Beyotime). To each cell suspension, 5 µL of Annexin V-FITC and 10 µL of PI were added. The resulting suspensions were gently vortexed and incubated at room temperature in the dark for 15 min. After the incubation, 400 µL of the binding buffer was added to each suspension, and the obtained mixtures were immediately analyzed using a flow cytometer. Fluorescence signals were detected in the FITC (Annexin V-FITC) and PE (PI) channels. Data were collected for 10,000 events per sample, and the cell populations were quantified.

**Mitochondrial membrane potential assessment**

4T1 cells were seeded in 6-well plates at a density of 4.0 × 10^5^ cells per well and cultured overnight. The cells were then treated for 6 h with four treatment formulations: (1) PBS (control), (2) AQ4N (200 μg/mL), (3) BPNSs (200 μg/mL), and a combination of BPNSs (200 μg/mL) and AQ4N (200 μg/mL). After the treatment, the culture medium was removed, and the cells were incubated with a freshly prepared JC-1 working solution from the enhanced mitochondrial membrane potential assay kit (C2006, Beyotime) at 37 °C for 40 min. The cells were then washed twice with PBS. Finally, fluorescence images were recorded for analysis.

**DNA damage assessment**

To assess DNA damage, 4T1 cells were passaged and seeded onto 18 × 18 mm glass coverslips (FCGF18, Beyotime) in 6-well plates at a density of 1.0 × 10^5^ cells per well and cultured overnight. The cells were then treated for 6 h with four treatment formulations: (1) PBS (control), (2) AQ4N (200 μg/mL), (3) BPNSs (200 μg/mL), and (4) a combination of BPNSs (200 μg/mL) and AQ4N (200 μg/mL). After the incubation, the cells were fixed with 4% paraformaldehyde for 15 min at room temperature, permeabilized with 0.1% Triton X-100 for 10 min, and washed with PBS. The cells were then blocked with 5% BSA for 1 h and incubated with the phospho-histone H2A.X (Ser139) rabbit polyclonal antibody (AF5836, Beyotime) overnight at 4 °C. Subsequently, the cells were incubated with a fluorophore-conjugated secondary antibody for 1 h at room temperature in the dark. Finally, the cell nuclei were stained with DAPI (1 μg/mL), and fluorescence images were recorded.

**Adenosine triphosphate assay**

Intracellular ATP levels were quantified using an enhanced ATP assay Kit (S0027, Beyotime). In brief, 4T1 cells were seeded in 6-well plates at a density of 2.0 × 10^4^ cells per well and cultured for 24 h. The cells were then treated for 12 h with four treatment formulations: (1) PBS (control), (2) AQ4N (200 μg/mL), (3) BPNSs (200 μg/mL), and (4) a combination of BPNSs (200 μg/mL) and AQ4N (200 μg/mL). After the treatment, the cells were washed once with cold PBS and lysed with the kit-provided lysis buffer (200 μL). The resulting lysates were collected and centrifuged (12000 rpm, 5 min) at 4 ℃ to remove cell debris. The supernatants were then transferred to new tubes and kept on ice until measurement. To quantify ATP, 100 μL of the ATP detection working solution from the kit was added to each well of a white 96-well plate, followed by the immediate addition of the obtained supernatant (20 μL). The plate was gently shaken, and the chemiluminescence intensity was measured immediately.

**F-actin cytoskeleton staining**

In a typical experiment, 4T1 cells were passaged and seeded onto 18 × 18 mm glass coverslips (FCGF18, Beyotime) in 6-well plates at a density of 1.0 × 10^5^ cells per well and cultured overnight. The cells were then treated for 12 h with either PBS (control) or a combination of BPNSs (200 μg/mL) and AQ4N (200 μg/mL). After the treatment, the cells were washed with PBS, fixed with 4% paraformaldehyde for 15 min at room temperature, permeabilized with 0.1% Triton X-100 for 10 min, and blocked with 5% BSA for 30 min. Next, the cells were incubated with an anti-F-actin primary antibody (ab205, Abcam) overnight at 4 °C, followed by an incubation with a fluorophore-conjugated secondary antibody for 1 h at room temperature in the dark. Finally, the cell nuclei were stained with DAPI (1 μg/mL), and fluorescence images were recorded.

**Wound-healing assay**

A wound healing (scratch) assay was performed to evaluate the migration ability of 4T1 cells. Briefly, 4T1 cells were seeded in 6-well plates at a density of 1.0 × 10^5^ cells per well and cultured overnight to form a confluent monolayer. A straight linear scratch was then created across the cell monolayer using a sterile pipette tip. Detached and floating cells were carefully removed by washing with PBS. Next, the cells were cultured in fresh medium containing PBS (control) or a combination of BPNSs (200 μg/mL) and AQ4N (200 μg/mL). Images of the scratched area were recorded at 0, 12, 24, and 36 h to monitor the wound closure. Representative images at 0 and 36 h were presented in the manuscript. ImageJ software was used to quantify the wound width, and relative migration was expressed as a percentage of wound closure compared to the initial wound width.

**In vivo experiments**

**Animal experiments**

To establish a subcutaneous tumor model, 4T1 cells in PBS (100 μL with a density of ~1 × 10^6^) were injected into the right flank of each mouse. The obtained 4T1 tumor-bearing mice were randomly divided into four groups (n = 6) when the tumor volume reached 100-120 mm^3^: (G1) a control group (intravenous (i.v.) injection of PBS), (G2) an AQ4N monotherapy group (i.v. injection, 4 mg/kg), (G3) a BPNSs monotherapy group (intratumoral (i.t.) injection, 4 mg/kg), and (G4) a BPNSs + AQ4N combined therapy group (i.t. injection of 4 mg/kg BPNSs and i.v. injection of 4 mg/kg AQ4N). For the combination therapy, BPNSs were first administered via i.t. injection, followed by an i.v. injection of AQ4N through the tail vein. Tumor volumes and body weights were measured every two days and recorded throughout the treatment period. Tumor volume was calculated using the standard formula (V = 0.5 × (length) × (width)^2^).

**Tumor staining**

**pH assessment.** To investigate the pH of tumors, two probes, BCECF-AM and SNARF 1, were used. Time-related intratumoral pH after the administration of BPNSs was studied using BCECF-AM. Generally, after the 4T1 tumor-bearing mice received an intratumoral injection of BPNSs (4 mg/kg), the tumors were harvested at 0, 3, 12, and 24 h post-injection and immediately embedded. Fresh-frozen tumor tissues were then sectioned at a thickness of 10 μm. The obtained tumor sections were incubated with BCECF-AM (10 μM, Beyotime, C2005) for 30 min at 37 °C in the dark, gently washed with PBS, and fixed with 4% paraformaldehyde for 15 min. Finally, the cell nuclei were stained with DAPI (1 μg/mL) for 10 min, and fluorescence images were recorded.

To compare the intratumoral pH differences between the control (G1) and the combined therapy (G4) groups, tumors from each group were collected. Fresh-frozen tumors were then sectioned at a thickness of 10 μm. The obtained tumor sections were incubated with SNARF 1 (10 μM, MCE, HY-D1021) for 30 min at 37 °C in the dark, gently washed with PBS, and fixed with 4% paraformaldehyde for 15 min. Finally, fluorescence images were recorded.

**Staining of mitochondrial calcium uniporter and hypoxia-inducible factor-1α.** Briefly, after the mice were treated, tumors were harvested from the mice in the control (G1) and the combined therapy (G4) groups. The obtained tumors were fixed in 4% paraformaldehyde for 24 h, embedded, and sectioned at a thickness of 10 μm. The resulting tumor sections were permeabilized with 0.3% Triton X‑100 for 10 min and blocked with 5% BSA for 1 h at room temperature. The sections were then incubated overnight at 4 °C with anti-MCU (14997, CST) and anti-HIF‑1α (ab179483, Abcam) antibodies for MCU and HIF-1α staining, respectively. After these sections were washed with PBS three times, they were incubated with fluorophore‑conjugated secondary antibodies for 1 h at room temperature in the dark. For the HIF‑1α-stained sections, the cell nuclei were stained with DAPI (1 μg/mL) for 10 min at room temperature. Finally, fluorescence images were recorded.

**Histopathological analysis**

At the end of the treatment, the mice were sacrificed. Major organs (spleen, liver, heart, kidney, and lung) and tumors were harvested and fixed with 4% paraformaldehyde for 24 h. For hematoxylin and eosin (H&E) staining, the fixed tissues were embedded in paraffin, sectioned at a thickness of 4 μm, and stained with hematoxylin and eosin following standard protocols. Finally, the slides were examined under a microscope.

To evaluate the tumor cell proliferation, the MKI67 (Ki-67) immunohistochemical assay was performed on the tumor sections. Briefly, the paraffin-embedded tumor tissues were deparaffinized in xylene, rehydrated, and subjected to antigen retrieval in citrate buffer (pH = 6.0) using a microwave for 10 min. After blocking endogenous peroxidase activity and nonspecific binding, the sections were incubated with a Ki-67 rabbit monoclonal antibody (28074-1-Ap, Proteintech) overnight at 4 °C. The corresponding HRP-conjugated secondary antibody was then applied, followed by chromogenic detection using 3,3′-diaminobenzidine. Finally, the cell nuclei were stained with hematoxylin, and the stained sections were examined under a microscope.

**Blood analysis**

To analyze the blood, fresh whole blood samples were collected from the mice at the end of the treatment period. The collected blood was drawn into anticoagulant tubes containing ethylenediaminetetraacetic acid and analyzed using an automated blood cell analyzer (BC-2800Vet, Mindray, China) for hematological analysis.

For serum biochemical analysis, whole blood was collected in serum-separating tubes and allowed to clot at room temperature for 1 h. The collected samples were then centrifuged (3000 rpm, 10 min) to obtain the serum. The biochemical parameters were evaluated using a fully automated biochemical analyzer (PBC22A Plus, LWPOCT).

All measurements were performed according to the standard protocols provided by the reagent kits and the equipment manufacturers.


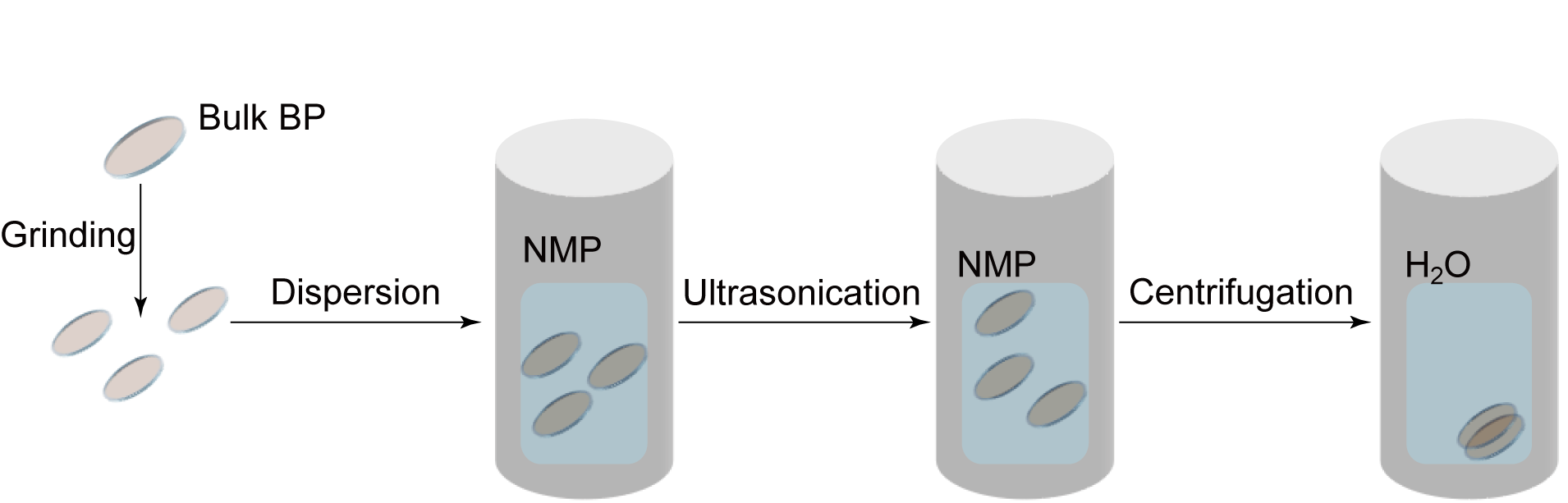


**Figure S1.** Schematic illustration of the synthesis of BPNSs.


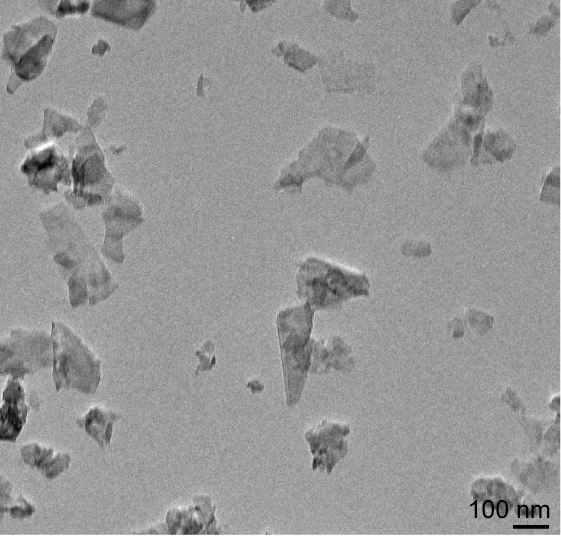


**Figure S2.** TEM image of the obtained BPNSs.

**
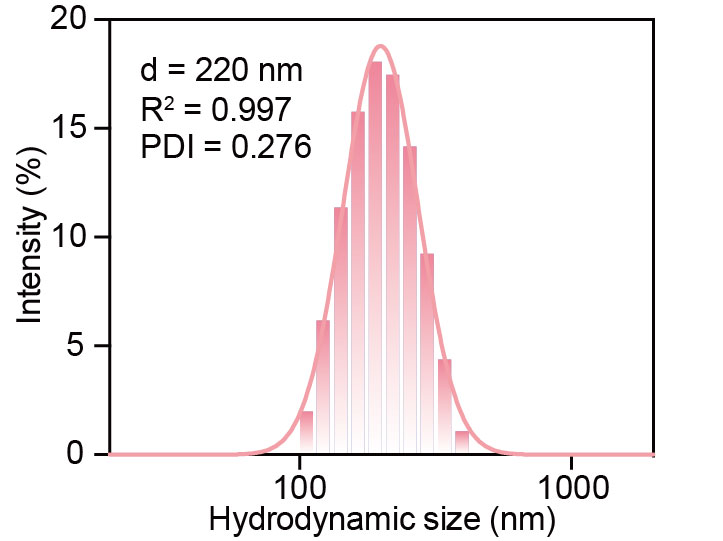
**

**Figure S3.** DLS analysis of BPNSs dispersed in ethanol.


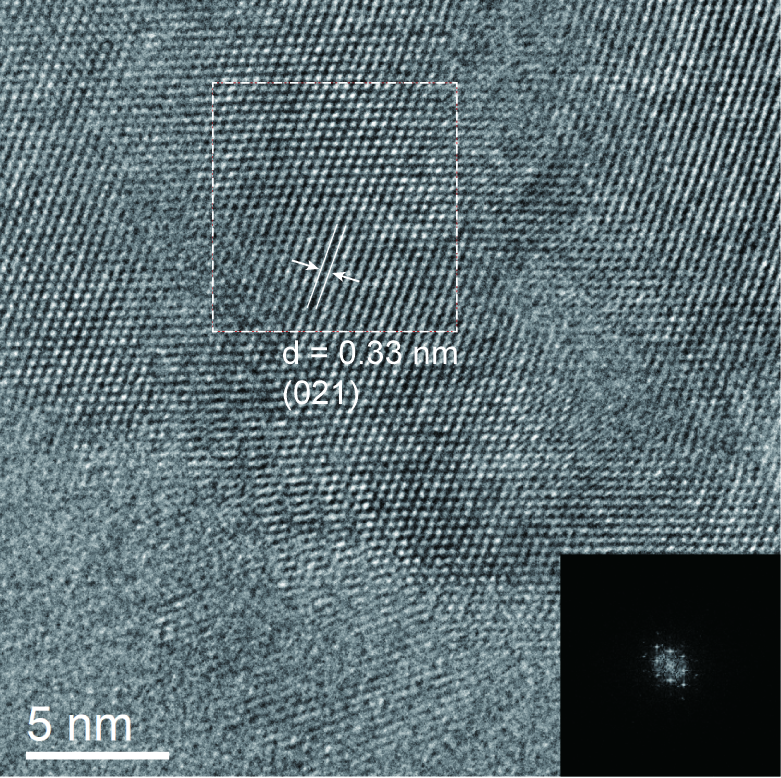


**Figure S4.** High-resolution TEM image of a BPNS. Inset is the fast Fourier transform image of the area indicated by the dashed rectangle in the TEM image.

**
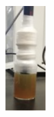
**

**Figure S5.** Photo of a sealed aqueous dispersion of BPNSs for detecting O_2_ consumption.


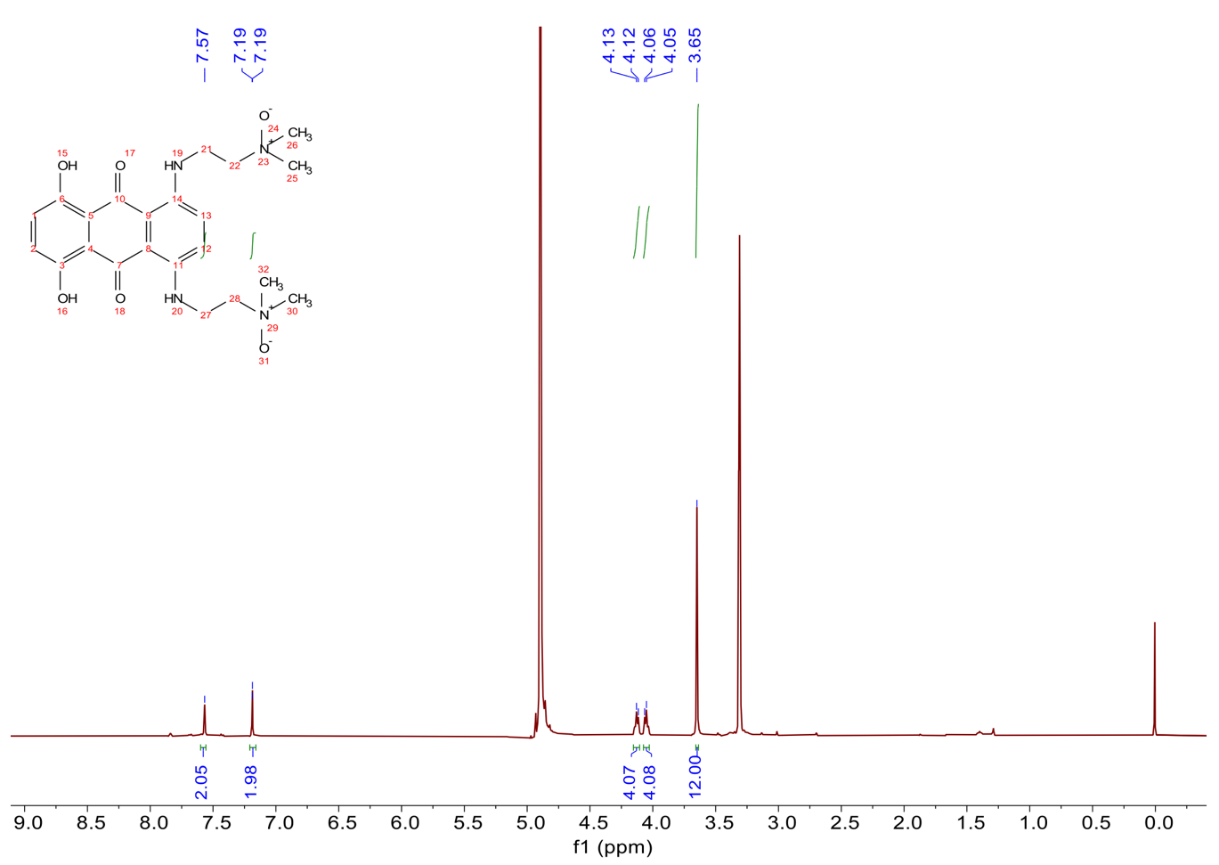


**Figure S6.** ^1^H NMR spectrum of AQ4N.


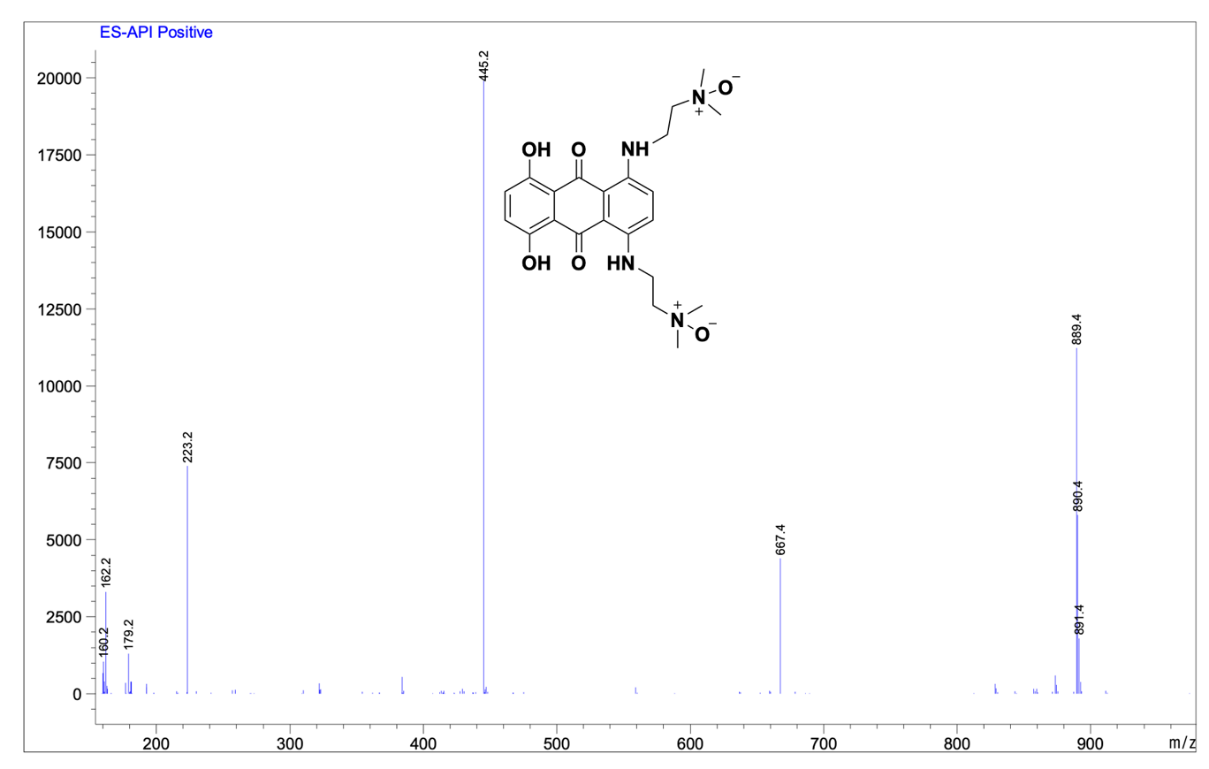


**Figure S7.** Screenshot of the mass spectrum of AQ4N (*m/z* 445). Inset is the chemical structure of AQ4N.


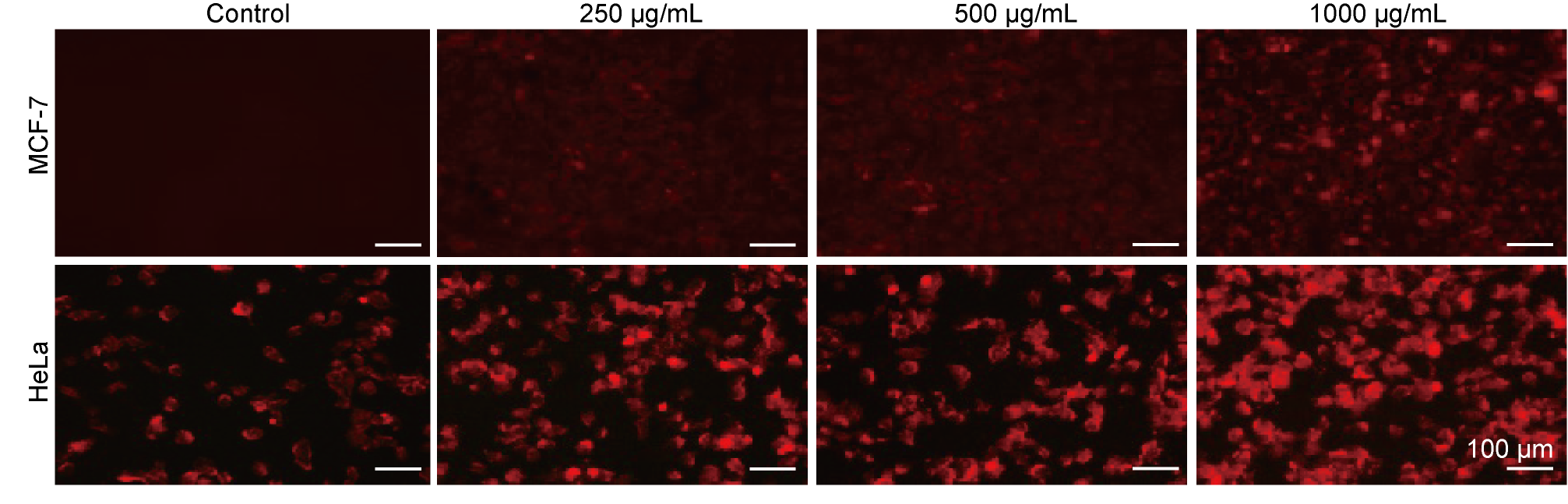


**Figure S8.** Fluorescence images showing the intracellular O_2_ level in MCF-7 and HeLa cells treated with different concentrations of BPNSs. Cells were stained with RDPP whose fluorescence intensity would be enhanced by the decrease of O_2_ in cells.


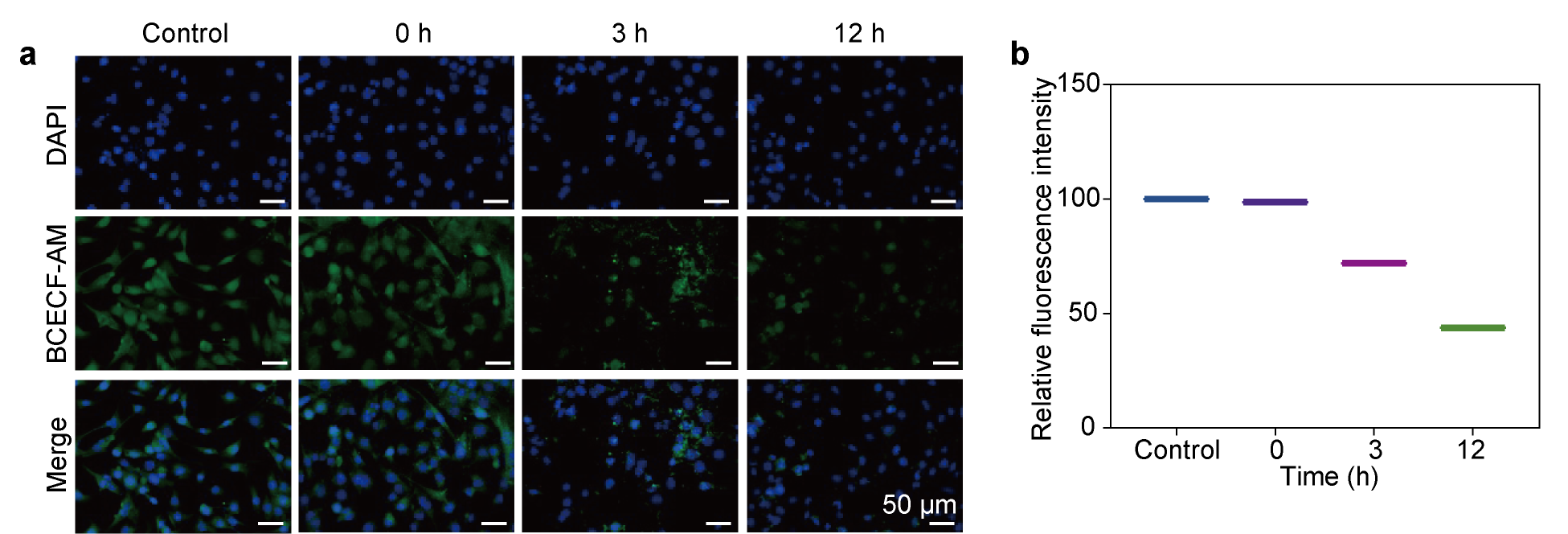


**Figure S9.** (a) Fluorescence images same as Figure 5d showing pH changes in 4T1 cells incubated with and without (control) BPNSs over time. Cell nuclei were stained with DAPI. (b) Corresponding fluorescence intensity of the BCECF-AM probe shown in (a). The fluorescence intensity of the control group was used as the reference.

**
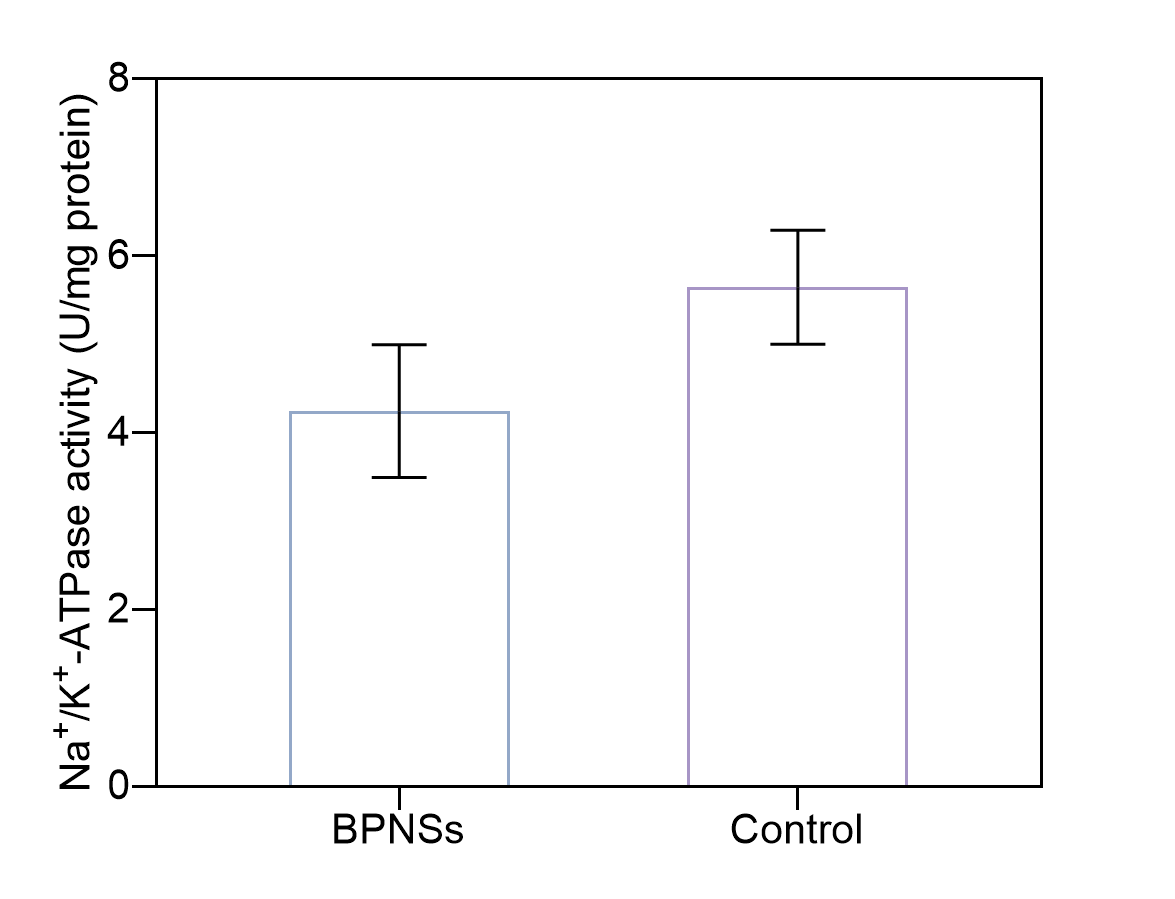
**

**Figure S10.** Na^+^/K^+^ adenosine triphosphatase (Na^+^/K^+^-ATPase) activity of cells incubated with and without (control) BPNSs, (mean ± SD, n = 3).


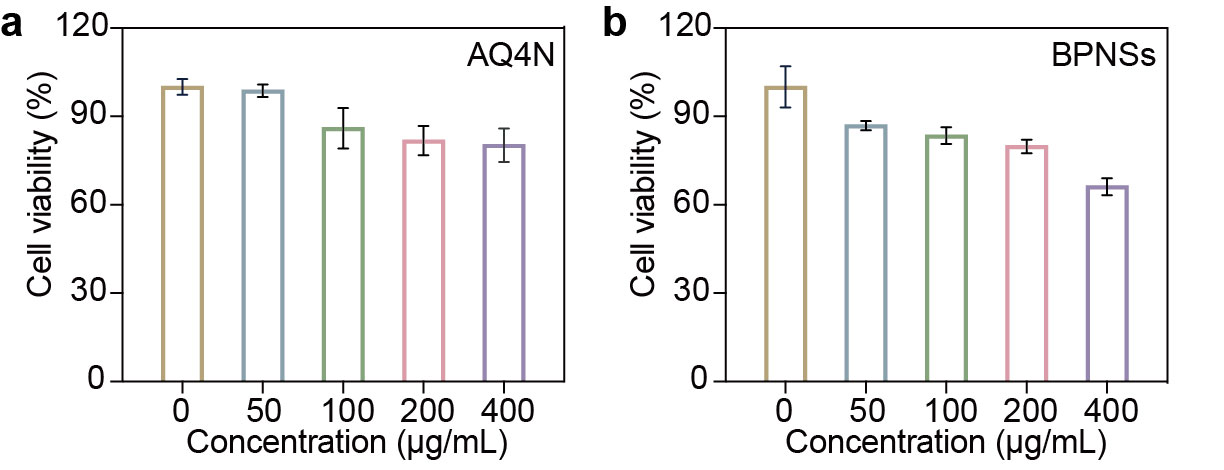


**Figure S11.** Cell viability of the 4T1 cells treated with various concentrations (0-400 μg/mL) of (a)

AQ4N and (b) BPNSs, (mean ± SD, n = 3).


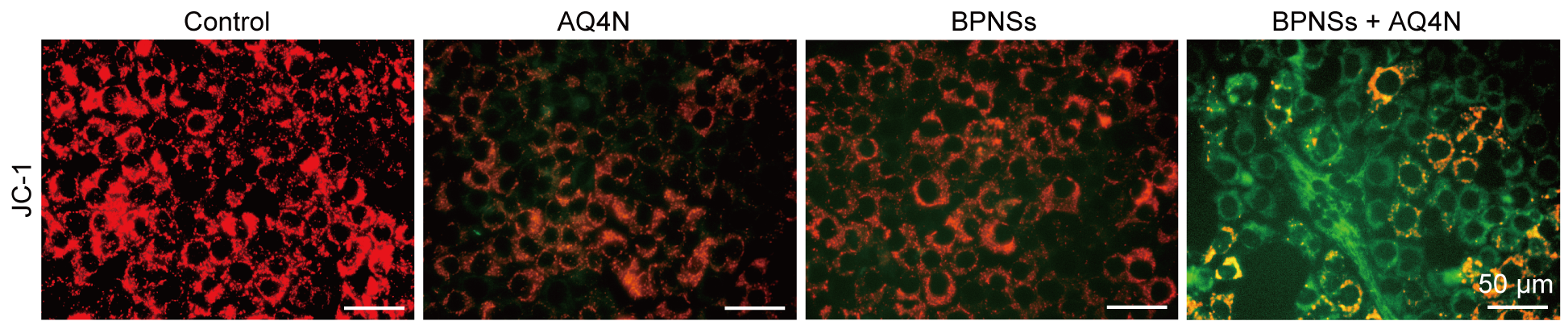


**Figure S12.** Fluorescence images of 4T1 cells stained by JC-1 probe after various treatments.


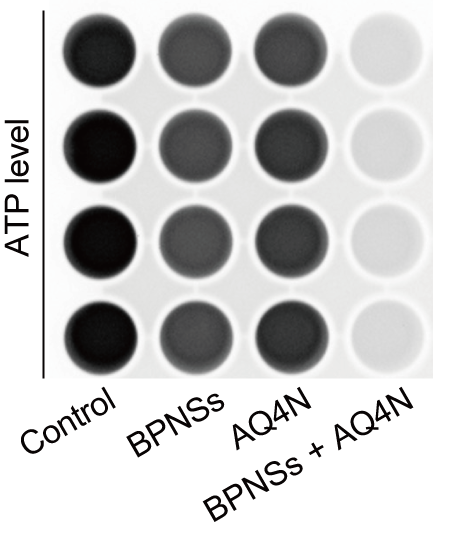


**Figure S13.** Measured ATP levels in cells treated with four treatment formulations (n = 4).

**
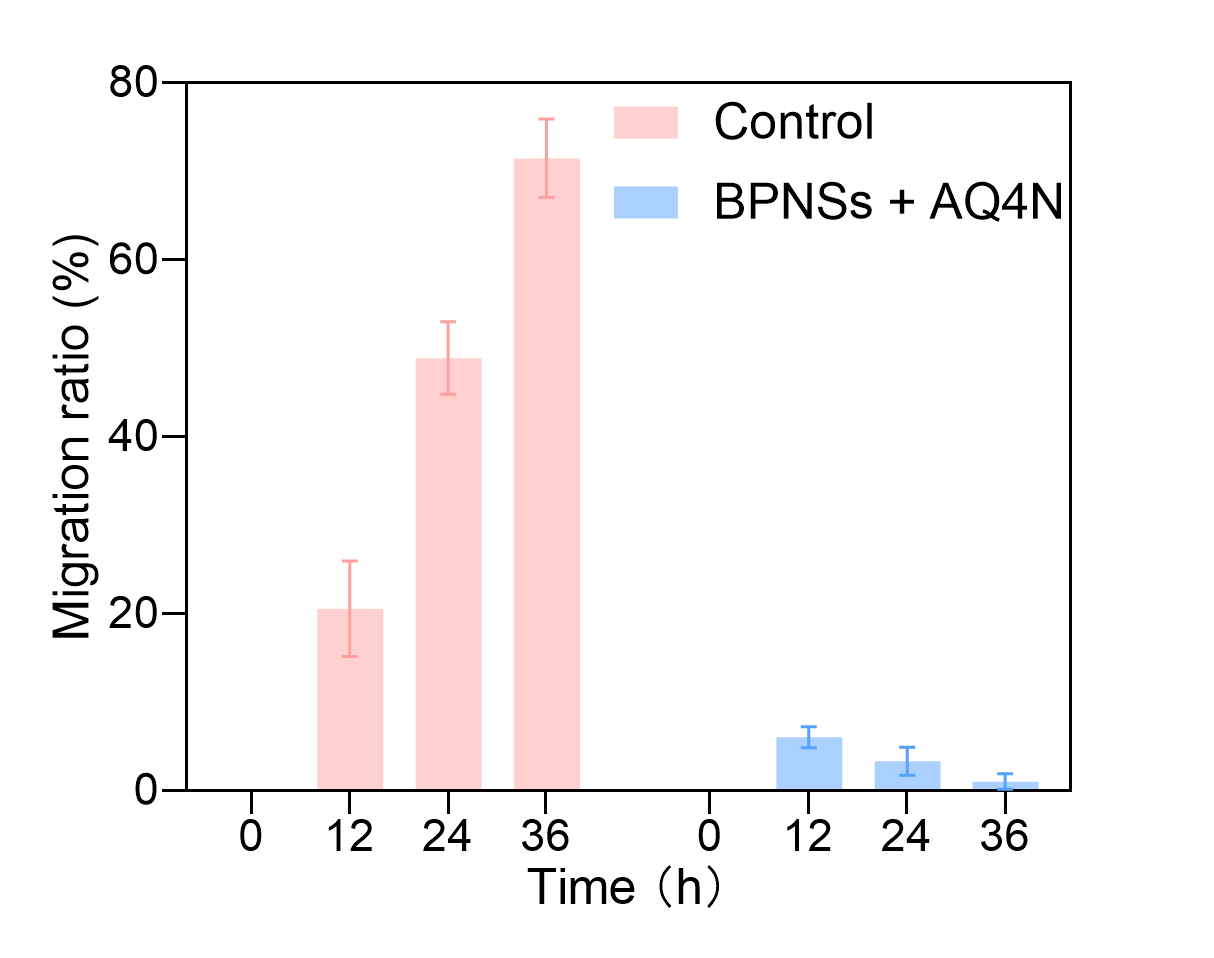
**

**Figure S14.** Cells migration ratios of untreated (control) and BPNSs + AQ4N-treated cells at different time points, (mean ± SD, n = 3).


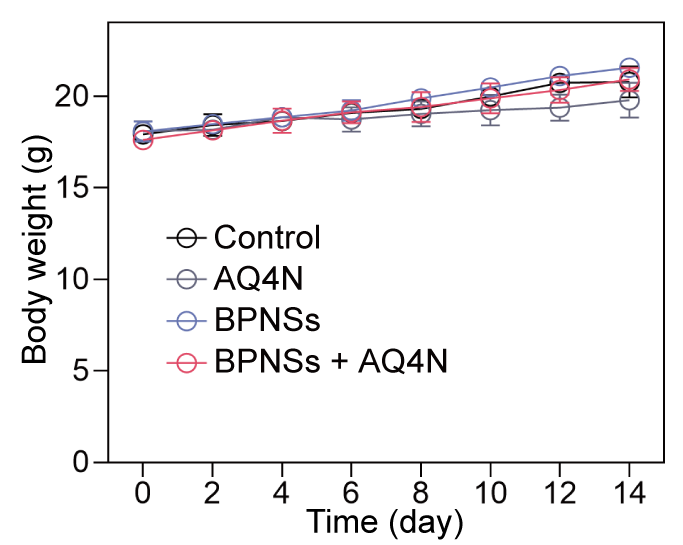


**Figure S15.** Changes in body weight of mice in different treatment groups.


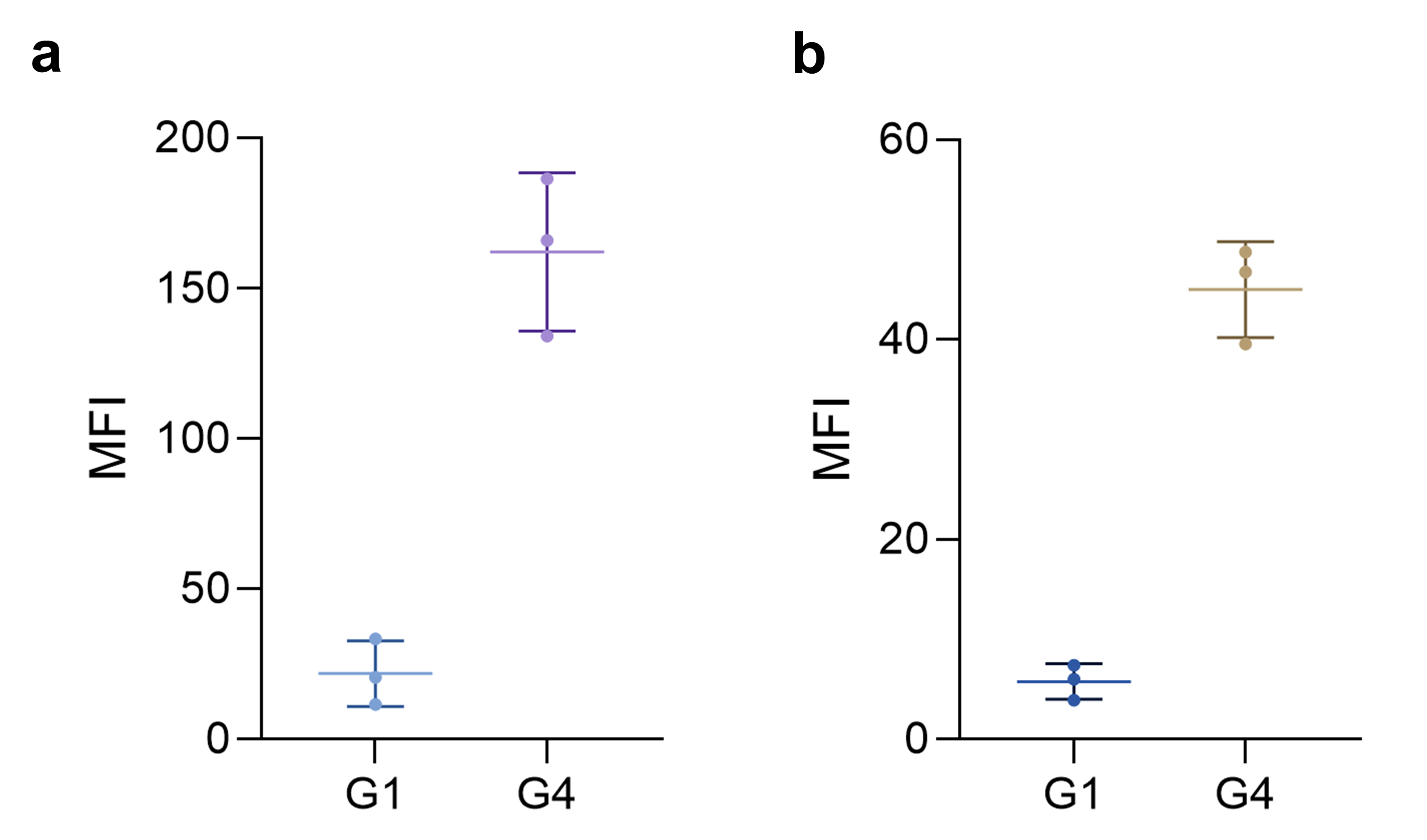


**Figure S16.** Corresponding mean fluorescence intensity (MFI) shown in (a) Figure 7f and (b) Figure 7h, (mean ± SD, n = 3).


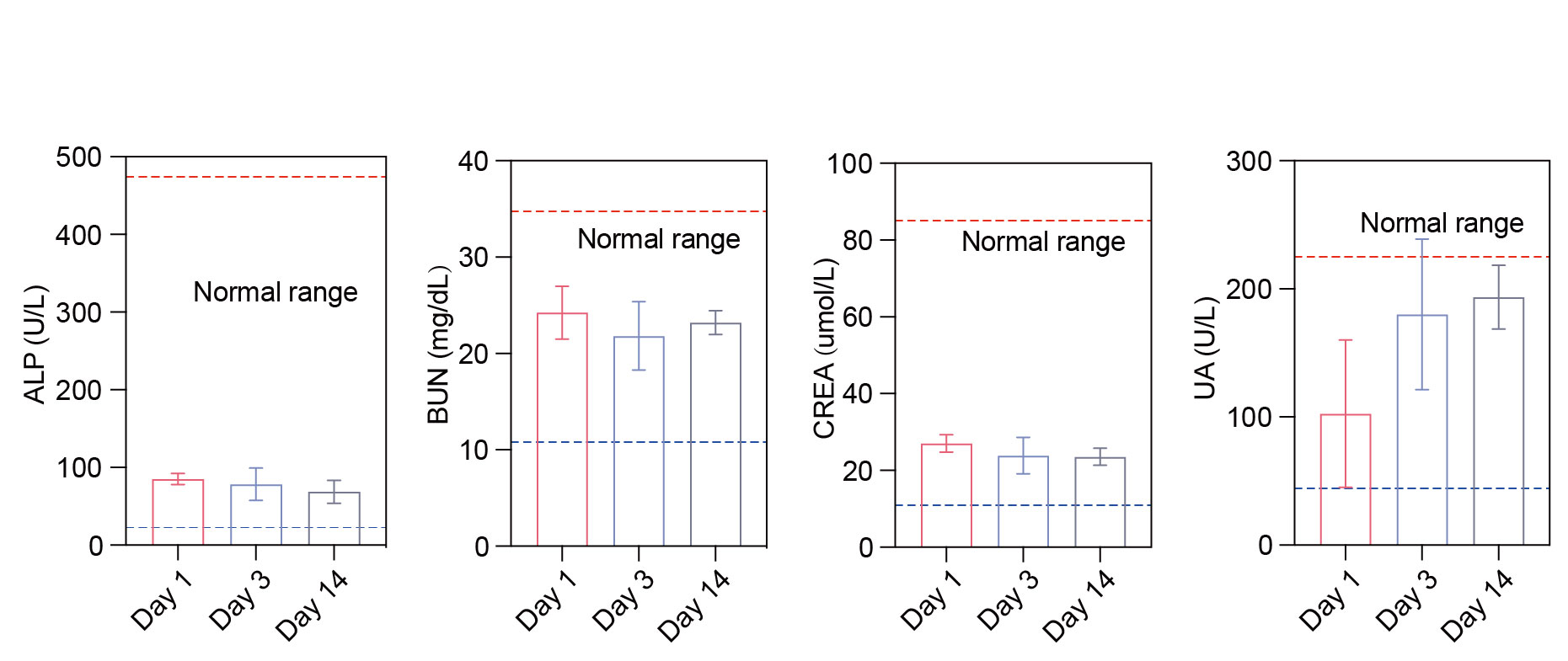


**Figure S1****7.** Serum biochemical indices measured in mice treated with BPNSs + AQ4N on days 1, 3, and 14 (ALP: alkaline phosphatase; BUN: blood urea nitrogen; CREA: creatinine; UA: uric acid). Blue and red dashed lines indicate the lower and upper limits of the normal range, respectively, (mean ± SD, n = 3).

**
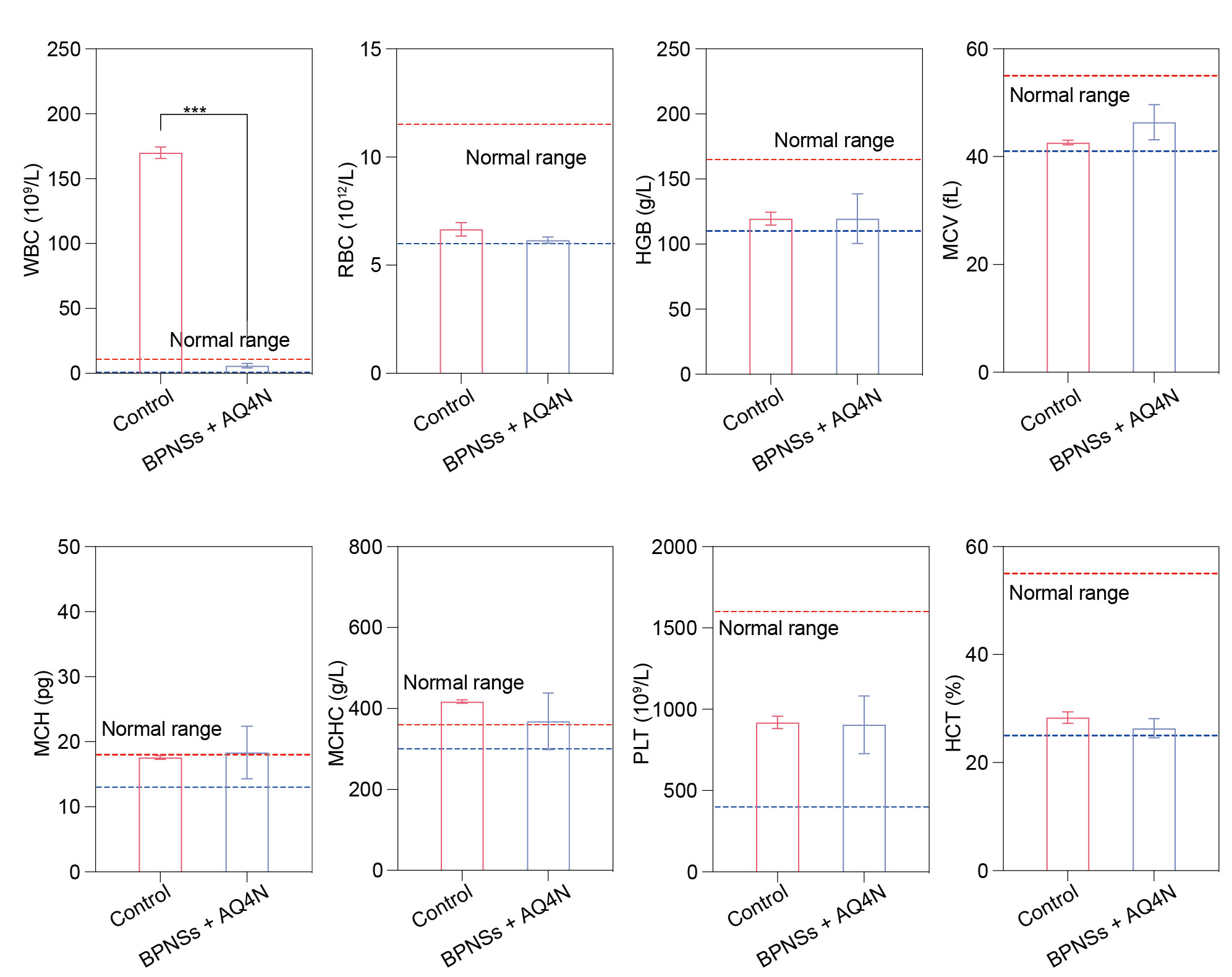
**

**Figure S18.** Whole blood analyses of mice treated with PBS (control) or BPNSs + AQ4N (WBC: white blood cells; RBC: red blood cells; HGB: hemoglobin; MCV: mean corpuscular volume; MCH: mean corpuscular hemoglobin; MCHC: mean corpuscular hemoglobin concentration; PLT: platelets; HCT: hematocrit). Blue and red dashed lines indicate the lower and upper limits of the normal range, respectively, (mean ± SD, n = 3).


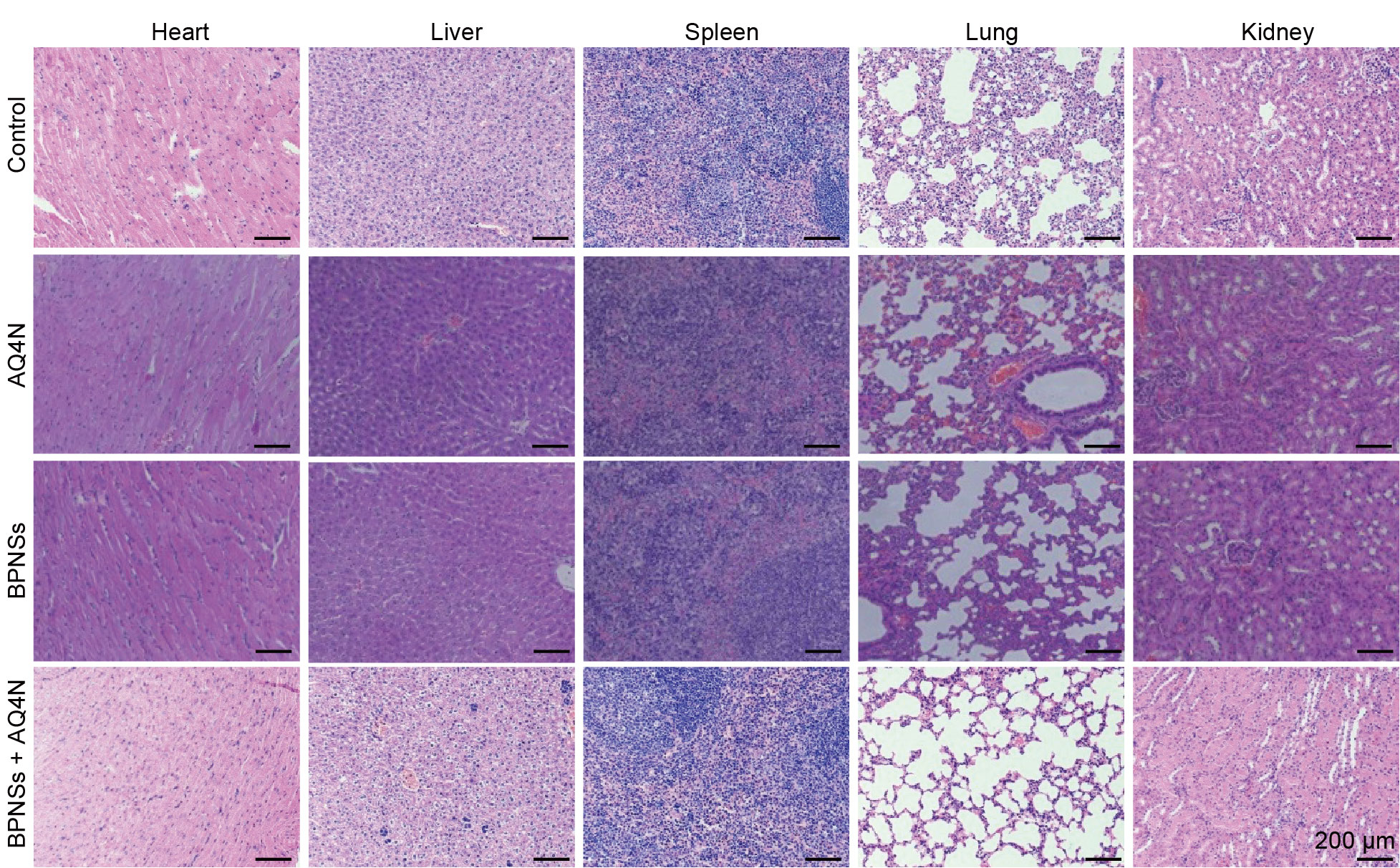


**Figure S19.** H&E staining of main organs, including heart, liver, spleen, lung, and kidney, collected from mice treated with PBS (control), AQ4N alone, BPNSs alone, and BPNSs + AQ4N, respectively.
